# Neonatal Unit Colonization Pressure and Acquisition of Extended-Spectrum β-Lactamase Gut Colonization

**DOI:** 10.1001/jamanetworkopen.2026.32644

**Published:** 2026-09-15

**Authors:** Aislinn Cook, Matilda Berkell, C. Henri van Werkhoven, Elske Sieswerda, David R. M. Smith, Chiara Minotti, Liesbet van Heirstraeten, Elias Iosifidis, Emmanuel Roilides, Tuuli Metsvaht, Jose Ignacio Pijoan Zubizarreta, Begoña Loureiro-Gonzalez, Eric Giannoni, Chiara Messina, Surbhi Malhotra-Kumar, Julia Anna Bielicki, and the NeoIPC Feasibility Group, Jennifer Martin, Filip Djukic, Caridad Tapia-Collados, Eva García-Cantó, Despoina Gkentzi, Eleni Papachatzi, Varvara Dimopoulou, Charles C. Roehr, Paula Brock, Eleftheria Hatzidaki, Nicolina-Hilda Anagnostatou, Eugenio Baraldi, Elena Priante, Kosmas Sarafidis, Angeliki Kontou, Georgios Mitsiakos, Dimitra Gialamprinou, Vassiliki Papaevangelou, Maria Livieratou, Kristin Tanney, Nicola Booth, Donna Tolentino, Amy Reid, Argyro Ftergioti, Maria Simitsopoulou, Kassandra Tataropoulou, Evangelia Koemtzidou, Concepción de Alba Romero, Elena Bergon Sendin, Javier Perez-Lopez, Yolanda Campos-Franco, Aleksandra Pietrzyk, Grete Haube, Mari-Liis Ilmoja, Helgi Padari, Martin Stocker, Alexandra Périsset, Dariusz Gruszfeld, Paul Clarke, Jasmine Myhill, Fernando Cabañas, Mario Giuffrè, Veronica Notarbartolo, Sven Wellmann, Anna Badura

**Affiliations:** 1 Centre for Neonatal and Paediatric Infection, City St George’s, University of London, London, United Kingdom; 2 Laboratory of Medical Microbiology, Vaccine and Infectious Disease Institute, Universiteit Antwerpen, Antwerp, Belgium; 3 Julius Center for Health Sciences and Primary Care, University Medical Center Utrecht, Utrecht University, Utrecht, the Netherlands; 4 European Clinical Research Alliance on Infectious Diseases (Ecraid), Utrecht, the Netherlands; 5 Epidemiology and Modelling of Antibiotic Evasion Unit, Institut Pasteur, Université Paris Cité, Paris, France; 6 Anti-Infective Evasion and Pharmacoepidemiology Team, Inserm U1018, CESP, UVSQ, Université Paris-Saclay, Montigny-le-Bretonneux, France; 7 Paediatric Research Centre, University Children’s Hospital Basel, Basel, Switzerland; 8 Department of Clinical Research, University of Basel, Basel, Switzerland; 9 3rd Department of Pediatrics, Aristotle University School of Medicine, Hippokration General Hospital, Thessaloniki, Greece; 10 Department of Microbiology, Institute of Translational Medicine, University of Tartu, Tartu, Estonia; 11 Children’s Clinic, Tartu University Hospital, Tartu, Estonia; 12 Biobizkaia Health Research Institute, Barakaldo, Spain; 13 CIBER of Epidemiology and Public Health (CIBERESP), Madrid, Spain; 14 Neonatal Unit, Cruces University Hospital, Barakaldo, Spain; 15 Clinic of Neonatology, Department Woman-Mother-Child, Lausanne University Hospital and University of Lausanne, Switzerland; 16 Fondazione Penta ETS, Padova, Italy; 17 Neonatology Section, Dr Balmis University General Hospital, ISABIAL, Alicante, Spain; 18 Department of Paediatrics, Medical School, University of Patras, Greece; 19 Faculty of Health and Life Sciences, University of Bristol, Bristol, United Kingdom; 20 Newborn Care, Women’s and Children Division, North Bristol NHS Trust, Bristol, United Kingdom; 21 National Perinatal Epidemiology Unit, University of Oxford, Oxford, United Kingdom; 22 Department of Neonatology/Neonatal Intensive Care Unit, University Hospital of Heraklion, School of Medicine, University of Crete; Heraklion, Crete, Greece; 23 Department of Women’ and Children’s Health, University of Padua, Padua, Italy; 24 Institute of Paediatric Research "Città della Speranza", Padua, Italy; 25 First Department of Neonatology and Neonatal Intensive Care, Aristotle University of Thessaloniki, Ippokrateion General Hospital, Thessaloniki, Greece; 26 2nd Neonatal Department and NICU, Aristotle University of Thessaloniki, “Papageorgiou” Hospital, Thessaloniki, Greece; 27 Third Department of Pediatrics, National and Kapodistrian University of Athens, Athens, Greece; 28 NICU, Attikon University Hospital, Athens, Greece; 29 NICU, St Mary’s Hospital, Manchester University NHS Foundation Trust, United Kingdom; 30 Neonatal Intensive Care Unit, St George’s University Hospitals NHS Foundation Trust, London, United Kingdom; 31 NICU—2nd Department of Pediatrics, NKUA, Children’s Hospital of Athens P. and A. Kyriakou, Athens, Greece; 32 Hospital 12 Octubre, Madrid, Sprain; 33 Spanish Network in Maternal, Neonatal, Child and Developmental Health Research (RICORS-SAMID, RD24/0013/0002), Instituto de Salud Carlos III, Madrid, Spain; 34 Neonatal Unit. Cruces University Hospital, Barakaldo, Spain; 35 Department of Neonatology and Neonatal Intensive Care, Children’s Memorial Health Institute, Warsaw, Poland; 36 Clinic of Anaesthesiology and Intensive Care, Tartu University Hospital, Tartu, Estonia; 37 Clinic of Anaesthesiology and Intensive Care, East Tallinn Central Hospital, Tallinn, Estonia; 38 Clinic of Anaesthesiology and Intensive Care, Tallinn Children’s Hospital, Tallinn, Estonia; 39 Luzerner Kantonsspittal, Luzerne, Switzerland; 40 Kantonsspital Aarau, Aarau, Switzerland; 41 Norwich Medical School, University of East Anglia, Norwich, United Kingdom; 42 Neonatal Intensive Care Unit, Norfolk and Norwich University Hospitals NHS Foundation Trust, Norwich, United Kingdom; 43 Department of Pediatrics and Neonatology, Quironsalud Madrid University Hospital and Quironsalud San José Hospital, Madrid, Spain; 44 Head of Neonatology, Nursery and Neonatal Intensive Care Unit, University Hospital Policlinic Paolo Giaccone of Palermo, Palermo, Italy; 45 Department of Health Promotion, Mother and Child, Excellence Internal and Specialist Medicine “G. D’Alessandro,” University of Palermo, Palermo, Italy; 46 Neonatology, Nursery and Neonatal Intensive Care Unit, University Hospital Policlinic Paolo Giaccone of Palermo, Palermo, Italy; 47 Department of Neonatology, University Children’s Hospital Regensburg (KUNO), Hospital St Hedwig of the Order of St John of God, University of Regensburg, Regensburg, Germany

## Abstract

**Importance:**

Understanding how neonatal unit colonization pressure, the proportion of infants carrying resistant bacteria on a unit, is associated with antibiotic-resistant bacterial acquisition risk can inform the design and implementation of effective infection prevention and control (IPC) interventions.

**Objectives:**

To describe resistant bacterial gene prevalence in European neonatal units and to assess the association between unit-level extended-spectrum β-lactamase (ESBL) colonization pressure and infant-level ESBL acquisition.

**Design, Setting, and Participants:**

Multicenter study in 24 tertiary neonatal units in 8 European countries (Estonia, Greece, Germany, Italy, Poland, Spain, Switzerland, and the United Kingdom) using repeated cross-sectional surveys collecting stool samples from infants over 4 surveys with 4-, 7-, and 14-day intervals, analyzed using real-time quantitative polymerase chain reaction (PCR) between January 2022 and June 2024 as part of the NeoIPC project. All infants present on the neonatal unit at 8:00 am on the day of the survey were eligible; infants could contribute to multiple surveys.

**Exposure:**

ESBL colonization pressure at previous survey.

**Main Outcome and Measure:**

Incident infant gut colonization with ESBL-harboring bacteria (ESBL-colonized). Bayesian hierarchical logistic regression modeling was used to assess the association between colonization pressure and infant-level acquisition risk on the neonatal unit.

**Results:**

Overall, 943 infants (547 of 943 male [58.0%]; median [IQR] gestational age, 34 [30-38] weeks, and median [IQR] birth weight, 2130 [1254-3084] g) contributed to 1847 infant-survey observations. Stool samples were collected and PCR results obtained in 1448 of 1847 infant surveys (78.4%). The most common genes detected were those encoding ESBLs (205 of 1448 samples [14.2%]), with high unit-level variation. In the colonization acquisition analysis, we included 829 samples from 533 infants hospitalized since birth who were either newly admitted since or not colonized at the previous survey time point; 90 of 533 infants (16.9%) became ESBL-colonized. Their median (IQR) gestational age was 34 (30-37) weeks, and median (IQR) length of stay at survey was 14 (5-32) days. Colonization pressure was associated with increased odds of becoming ESBL-colonized for all survey intervals (4-day interval: mean odds ratio [OR], 1.89; 95% credible interval [CrI], 1.22-2.94; 7-day interval: OR, 1.76; 95% CrI, 1.26-2.63; 14-day interval: OR, 1.73; 95% CrI, 1.15-2.52 per 10% increase of colonization pressure).

**Conclusions and Relevance:**

In this cross-sectional study, ESBL colonization pressure was associated with individual risk of acquiring ESBL on the neonatal unit. Unit-level interventions targeting colonization rather than infection may have important direct and indirect benefits in units with high colonization pressure.

## Key Points

**Question:**

Is higher unit-level resistant bacterial colonization pressure associated with increased infant-level acquisition risk in European neonatal units?

**Findings:**

Using repeated cross-sectional colonization assessments in 24 European neonatal units, this study found that higher prevalence of infant colonization with extended-spectrum β-lactamase (ESBL)-harboring bacteria on the neonatal unit was associated with increased odds of an infant becoming ESBL-colonized, independent of prematurity, length of stay, prior antibiotic exposure, and unit effects.

**Meaning:**

These findings suggest that infants are at greater risk of acquiring ESBL-harboring bacteria on neonatal units with higher colonization pressure, highlighting the importance of unit-level mitigating infection prevention and control interventions.

## Teaser

This cross-sectional study evaluates the association between extended-spectrum β-lactamase (ESBL) colonization prevalence in European neonatal units and infant-level ESBL acquisition risk.

## Introduction

Invasive infections in neonates are associated with high mortality and morbidity, including long-term neurodevelopmental impairment, particularly among infants born preterm.^1,2,3^ Neonatal sepsis is the most common manifestation of these infections and is more difficult to treat when caused by resistant bacteria.^4^ Bacterial colonization is a recognized preceding step for invasive infection and is a source of transmission within health care settings.^5,6^

For hospitalized infants, the health care environment is a likely source of postnatal acquisition of resistant bacteria.^7,8,9^ Infants on neonatal units are immobile and exposed to shared equipment, health care workers, and the surrounding environment, facilitating bacterial transmission and acquisition.^10^

Infection prevention and control (IPC) on neonatal units aims to reduce transmission events and, in turn, avert invasive infection. Relevant studies examining risk factors for acquisition have focused on infant-level factors or combined infection and colonization outcomes, or on *Staphylococcus aureus*.^11,12,40^ Few studies of concurrently hospitalized individuals have assessed the associations between colonization status and acquisition risk, particularly with resistant Gram-negative bacteria. Colonization pressure, the proportion of colonized infants on the unit, is associated with ongoing transmission of resistant bacteria in other intensive care settings,^13,14,15,16,17^ but evidence on its association with acquisition of resistant Gram-negative bacterial colonization in neonates is limited.^18,19,20^

A better understanding of the association between colonization pressure and individual incident colonization risk is needed to inform IPC strategies on neonatal units. We aimed to describe the prevalence of resistant bacterial colonization in hospitalized infants in neonatal units in Europe and to quantify the association between unit-level colonization pressure and individual risk of acquisition of extended-spectrum β-lactamase (ESBL)-harboring bacterial

## Methods

### Study Design

The NeoIPC Project^21^ evaluates and implements IPC measures to reduce resistant bacterial infections in neonatal units across Europe. As part of the design of an interventional IPC trial, NeoDeco (NCT05993442), we undertook a multicenter colonization assessment across 24 neonatal units in 8 European countries (Estonia, Germany, Greece, Italy, Poland, Spain, Switzerland, and the United Kingdom) between January 2022 and June 2024. The assessment aimed to measure resistant bacterial colonization prevalence among hospitalized infants and explore infant incident colonization dynamics. Neonatal units eligible for inclusion were those providing routine care of extremely premature babies (ie, those <28 weeks’ gestational age). Full site characteristics are in eTable 1 in Supplement 1.

### Data Collection

Participating neonatal units conducted 4 cross-sectional colonization surveys within a 4-week period. Within each unit, the intervals between surveys were 4, 7, and 14 days, with the order of intervals randomized between units. These intervals were selected primarily to look at differences in colonization pressure over time to inform trial sampling strategies; however, we exploit this variation to look at the association between colonization pressure and infant incident colonization.

At each survey, all infants present on the neonatal unit at 8 am on the day of sampling were eligible for inclusion, with no exclusion criteria. Stool samples and skin swabs were collected from all eligible infants on the unit unless the infant’s caregiver opted out, consistent with standard surveillance approaches. Parents and legal guardians were informed through written information sheets and ward-based notices and could opt out of data collection, sample collection, or both. All site ethics committees approved the opt-out approach to consent. Infants were assigned a random study ID number, which was retained across surveys for infants who remained hospitalized and contributed to multiple surveys. Clinical data collected included demographics, delivery and admission information, antibiotic exposure since admission, and postnatal age, length of stay, and relevant clinical risk factors at each survey.

Data were collected in REDCap^22,23^ a secure, web-based data capture system, hosted at City St. George’s, University of London. The protocol was approved by City St. George’s, University of London and by each participating hospital’s ethics committee before commencing the study. This study follows the Strengthening the Reporting of Observational Studies in Epidemiology (STROBE) reporting guidelines for cross-sectional studies.

### Microbiological Sampling and Analysis

All collected samples were shipped to the Laboratory of Medical Microbiology at University of Antwerp for targeted detection of resistance genes by real-time quantitative polymerase chain reaction (RT-qPCR); data were collected in ClinSLIMS. Assays targeted genes conferring antibiotic resistance (ARGs) relevant to bacteria commonly causing neonatal sepsis or listed as WHO priority pathogens.^24^ For stool samples, we determined presence of genes encoding carbapenem resistance (bla*_KPC_*, bla*_VIM_*, bla*_NDM_*, bla*_OXA-48_*, bla*_IMP_*), ESBLs (bla*_CTX-M,_* bla*_SHV_*), and vancomycin resistance (*vanA, vanB)* (eTable 2 in Supplement 1). For skin swabs, we focused on methicillin resistance genes (*mecA, mecC, SCCmec/orfX)* (eTable 3 in Supplement 1). A sample was considered positive when the cycle threshold (Ct) for the relevant gene and selected assay was less than 37. Samples positive for bla*_SHV_* were considered to indicate ESBL-presence only upon further testing (eMethods in Supplement 1). Colonization status of a given anatomical niche was considered unknown if the relevant sample was not collected or insufficient for analysis. Clinical and laboratory data were merged for analysis using R version 4.4.3 (R Project for Statistical Computing).

### Statistical Analysis

We analyzed the data from all infants and for all resistance genes descriptively, presenting frequency (number and percentage) for categorical variables and median (IQR) for continuous variables for the whole study population.

Our main analysis focused on the association between unit-level colonization pressure and infant acquisition of resistant bacteria on the neonatal unit. We planned to analyze all the gene types measured in the study, but due to low prevalence of these genes in only a few sites, this was not possible. For this analysis, the primary outcome was incident gut colonization with ESBL-harboring bacteria during neonatal unit stay, as these were the most common resistance genes detected across most units. Infants were considered ESBL-colonized if bla*_CTX-M_* group 1 or bla*_CTX-M_* group 9 were detected in stool samples. The primary exposure of interest was ESBL-colonization pressure, defined as the proportion of infants on the neonatal unit who were ESBL-colonized at the previous survey time point.

To focus on hospital acquisition for this analysis, we restricted the study population to infants born at the participating site and hospitalized from birth. We further limited inclusion to infants who were either not colonized at the previous survey or newly admitted since the previous survey. As previous colonization pressure was unknown at the first survey in each unit, acquisition was restricted to the second, third, and fourth surveys with colonization pressure exposure calculated from the first, second and third surveys, respectively (eFigure 1 in Supplement 1).

Because the time interval order between surveys varied by design (4, 7, or 14 days), in different order between hospitals, we constructed 3 separate model-fitting datasets, each corresponding to one of these interval lengths (eFigure 1 and eFigure 2 in Supplement 1). Each dataset included infants as described previously, with complete covariates and known ESBL status and whose exposure (colonization pressure) was calculated from the immediately preceding survey at the corresponding interval. These 3 datasets together constitute the main analysis and were analyzed separately to assess consistency of associations across different exposure windows.

Colonization pressure was scaled by a factor of 10 so a 1-unit change represented a 0.1 increase in the proportion of colonized infants. Length of stay (LOS) on neonatal unit was calculated as weeks to represent change in acquisition risk per week in the hospital.

We fit a bayesian hierarchical multivariable logistic regression model (R package *brms* version 2.22.0) with random intercepts for neonatal unit to account for clustering of infants and to account for unmeasured neonatal unit-level factors such as IPC practices. We selected variables for inclusion in the model a priori based on those collected and considered confounders for the association between colonization pressure and becoming colonized (not for becoming infected): LOS at the time of survey, prematurity category (born <32 weeks or ≥32 weeks gestation), and any antibiotic exposure in the previous 14 days or since admission (if LOS <14 days) (see eMethods and eFigure 3 in Supplement 1 for more information).

We used informative normal priors with a mean (SD) of 0 (1.5) for covariate coefficients, a *t* prior with 3 degrees of freedom (location, −2; scale, 1.5) for the intercept, and an exponential prior with a rate of 1 for the SD of the neonatal unit random intercept. Sensitivity to prior choice was assessed and did not materially affect posterior estimates (eTable 4, eFigure 4, and eFigure 5 in Supplement 1). Model fit was checked using posterior predictive checks to compare posterior predictive distribution and observed colonization outcome distribution (eMethods and eFigure 6 in Supplement 1).

Some units had no ESBL-colonized infants at any survey, resulting in colonization pressure of 0 throughout. To assess whether this had skewed the findings, we conducted a sensitivity analysis restricted to neonatal units that had at least 1 ESBL-colonized infant at all 4 survey time points. We refit the same 3 interval-specific models (4-, 7-, and 14-day datasets) using the same model structure and priors as in the primary analysis (eMethods in Supplement 1).

We report model coefficients as mean odds ratio (OR) with 95% credible interval (CrI) (the range for which 95% of the posterior distribution estimates falls into) and the probability of the direction of effect (representing the certainty of existence of the direction of the effect). ORs where the 95% CrI does not cross 1 were considered statistically significant. For standard deviation of neonatal unit random intercepts, we report mean and 95% CrI of the log-odds deviation from the global intercept.

## Results

### Overall Study Population

A total of 943 infants from 24 neonatal units contributed to 1847 infant-survey observations across 4 cross-sectional colonization surveys (Table 1 and eTable 5 in Supplement 1). A total of 547 infants (58.9%) were male, 594 of 943 (63.0%) were born via cesarean section, the median (IQR) gestational age was 34 (30-38) weeks, median (IQR) birth weight was 2130 (1254-3084) g, and 315 of 943 infants (33.4%) were born at less than 32 weeks’ gestation. A total of 469 of 943 infants (49.7%) contributed to 1 survey, 203 of 943 (21.5%) to 2 surveys, 112 of 943 (11.9%) to 3 surveys, and 159 of 943 (16.9%) to all 4 surveys.

**Table 1.** Characteristics of Population of Infants for All Infant Surveys for Gut ESBL Resistant Bacterial Colonization Status, Defined as Detection of ESBL Gene of Interest in Stool Sample at the Survey Time Point^a^

Characteristic	Overall (N = 1847)	Infant gut ESBL colonization status		
		Not colonized (n = 1243)	Colonized (n = 205)	Unknown (n = 399)^b^
Country				
Estonia	33 (1.8)	33 (2.7)	0	0
Germany	125 (6.8)	93 (7.5)	6 (2.9)	26 (6.5)
Greece	401 (21.7)	264 (21.2)	80 (39.0)	57 (14.3)
Italy	105 (5.7)	56 (4.5)	14 (6.8)	35 (8.8)
Poland	141 (7.6)	85 (6.8)	16 (7.8)	40 (10.0)
Spain	296 (16.0)	184 (14.8)	63 (30.7)	49 (12.3)
Switzerland	263 (14.2)	223 (17.9)	2 (1.0)	38 (9.5)
United Kingdom	483 (26.2)	305 (24.5)	24 (11.7)	154 (38.6)
Postnatal age at survey, median (IQR), d	19.0 (8.0-44.0)	19.0 (7.0-42.0)	23.0 (10.0-52.0)	19.0 (6.0-48.0)
Length of stay on NICU at survey, median (IQR), wk	2.0 (0.7-4.7)	2.0 (0.7-4.6)	2.3 (0.9-5.4)	1.6 (0.4-5.0)
Antibiotic exposure within 14 d				
No	809 (43.8)	546 (43.9)	109 (53.2)	154 (38.6)
Yes	1032 (55.9)	693 (55.8)	95 (46.3)	244 (61.2)
Unknown	6 (0.3)	4 (0.3)	1 (0.5)	1 (0.3)
Surgery before survey				
No	1424 (77.1)	970 (78.0)	169 (82.4)	285 (71.4)
Yes	403 (21.8)	259 (20.8)	36 (17.6)	108 (27.1)
Unknown	20 (1.1)	14 (1.1)	0	6 (1.5)
Skin-to-skin contact in prior 24 h				
No	767 (41.5)	484 (38.9)	91 (44.4)	192 (48.1)
Yes	977 (52.9)	695 (55.9)	112 (54.6)	170 (42.6)
Unknown	103 (5.6)	64 (5.1)	2 (1.0)	37 (9.3)
Enteral breast milk in prior 24 h				
No	505 (27.3)	290 (23.3)	57 (27.8)	158 (39.6)
Yes	1112 (60.2)	777 (62.5)	120 (58.5)	215 (53.9)
Unknown	230 (12.5)	176 (14.2)	28 (13.7)	26 (6.5)
Presence of central line within 24 h				
No	1416 (76.7)	990 (79.6)	174 (84.9)	252 (63.2)
Yes	428 (23.2)	251 (20.2)	31 (15.1)	146 (36.6)
Unknown	3 (0.2)	2 (0.2)	0	1 (0.3)
Receipt of TPN in prior 24 h				
No	1414 (76.6)	991 (79.7)	177 (86.3)	246 (61.7)
Yes	420 (22.7)	245 (19.7)	27 (13.2)	148 (37.1)
Unknown	13 (0.7)	7 (0.6)	1 (0.5)	5 (1.3)
Receipt of invasive ventilation within prior 24 h				
No	1656 (89.7)	1140 (91.7)	202 (98.5)	314 (78.7)
Yes	184 (10.0)	97 (7.8)	3 (1.5)	84 (21.1)
Unknown	7 (0.4)	6 (0.5)	0	1 (0.3)

Abbreviations: ESBL, extended-spectrum β-lactamase; NICU, neonatal intensive care unit; TPN, total parenteral nutrition.

^a^ Infants could contribute to more than 1 survey, therefore birth characteristics are not presented because they will overrepresent infants in more than 1 survey.

^b^ Unknown includes infants who did not produce a stool sample, were not available during sample collection, or where stool volume was too low for analysis.

Stool samples were collected with available PCR results for 1448 of 1847 infant surveys (78.4%); the most common reason for missing stool samples was the infant not passing stool in the survey time window. Skin swabs were collected and had available results for 1830 of 1847 infant surveys (99.1%).

Overall, 308 of 1448 stool samples (21.3%) were ARG-positive, of which ESBL genes, predominantly *bla*_CTX-M_ group 1, were most commonly detected (205 of 1448 stool samples [14.2%]), followed by carbapenemase genes (101 of 1448 stool samples [7.0%]) and vancomycin resistance genes (74 of 1448 stool samples [5.1%]) (eTable 6 and eTable 7 in Supplement 1). A total of 37 of 1830 skin swabs (2.0%) were positive for methicillin-resistance genes harbored by *Staphylococcus aureus*. ARGs primarily associated with methicillin-resistant coagulase-negative staphylococci, although not a primary target of surveillance, were detected in most skin swabs (1694 of 1830 swabs [92.6%]). Detailed microbiological findings are presented in eTable 7 and eTable 8 in Supplement 1.

**Figure 1.**
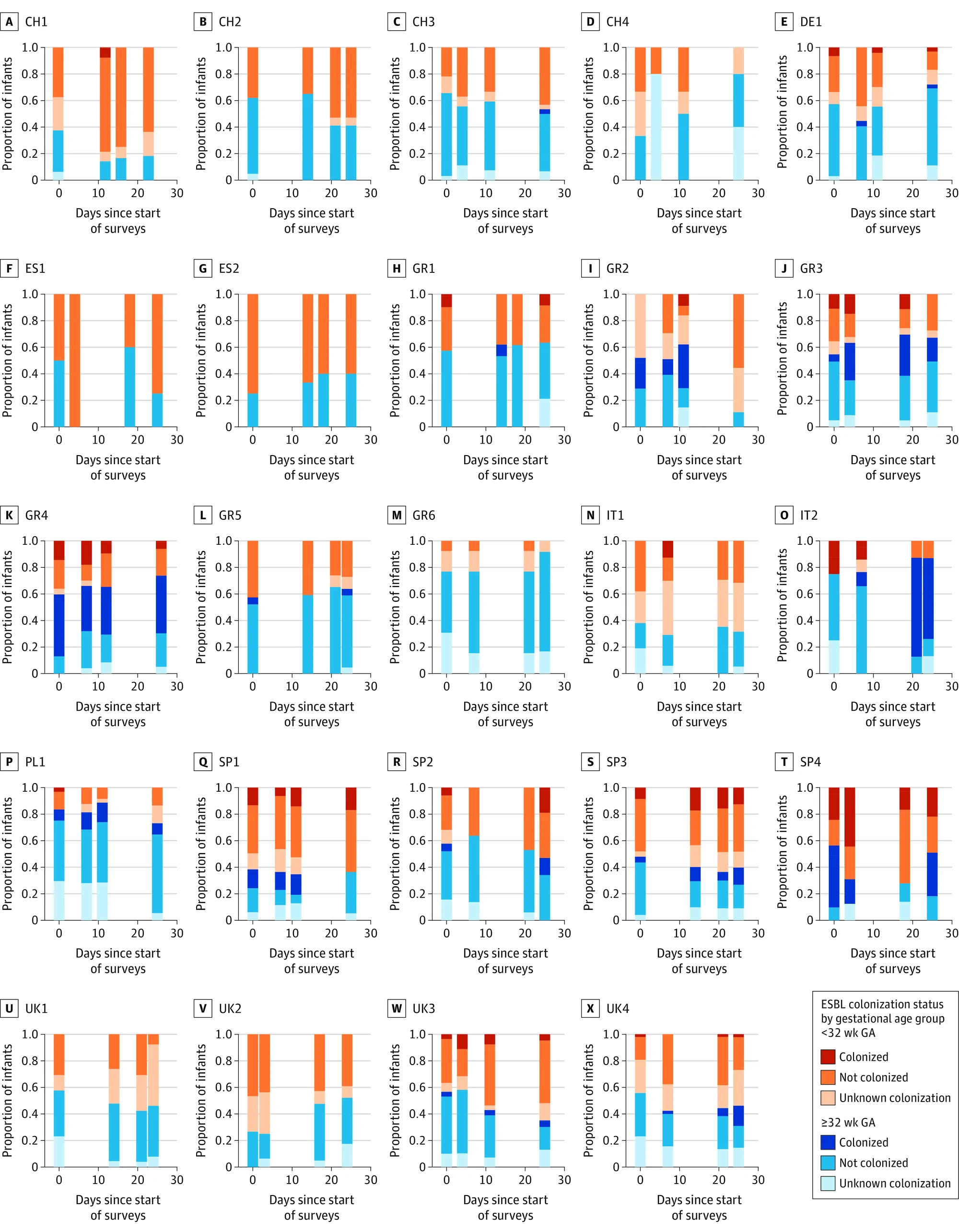
Proportion of Infants by Extended-Spectrum β-Lactamase (ESBL) Colonization Status and Prematurity Status, Both Plots Shown by Unit and by Days Since the Start of the Survey Period Illustrating the Gaps Between Surveys GA indicates gestational age. Site names represent country names: CH, Switzerland; DE, Germany; ES, Estonia; GR, Greece; IT, Italy; PL, Poland; SP, Spain; UK, United Kingdom.

The proportion of infants colonized with ESBL genes varied between sites (0%-39.0%) (Table 1 and eFigure 7 in Supplement 1). Where ESBL genes were detected, both infants born before or after 32 weeks’ gestation were found to be colonized across sites and survey time points (Figure 1).

### Colonization Acquisition Analysis

**Figure 2.**
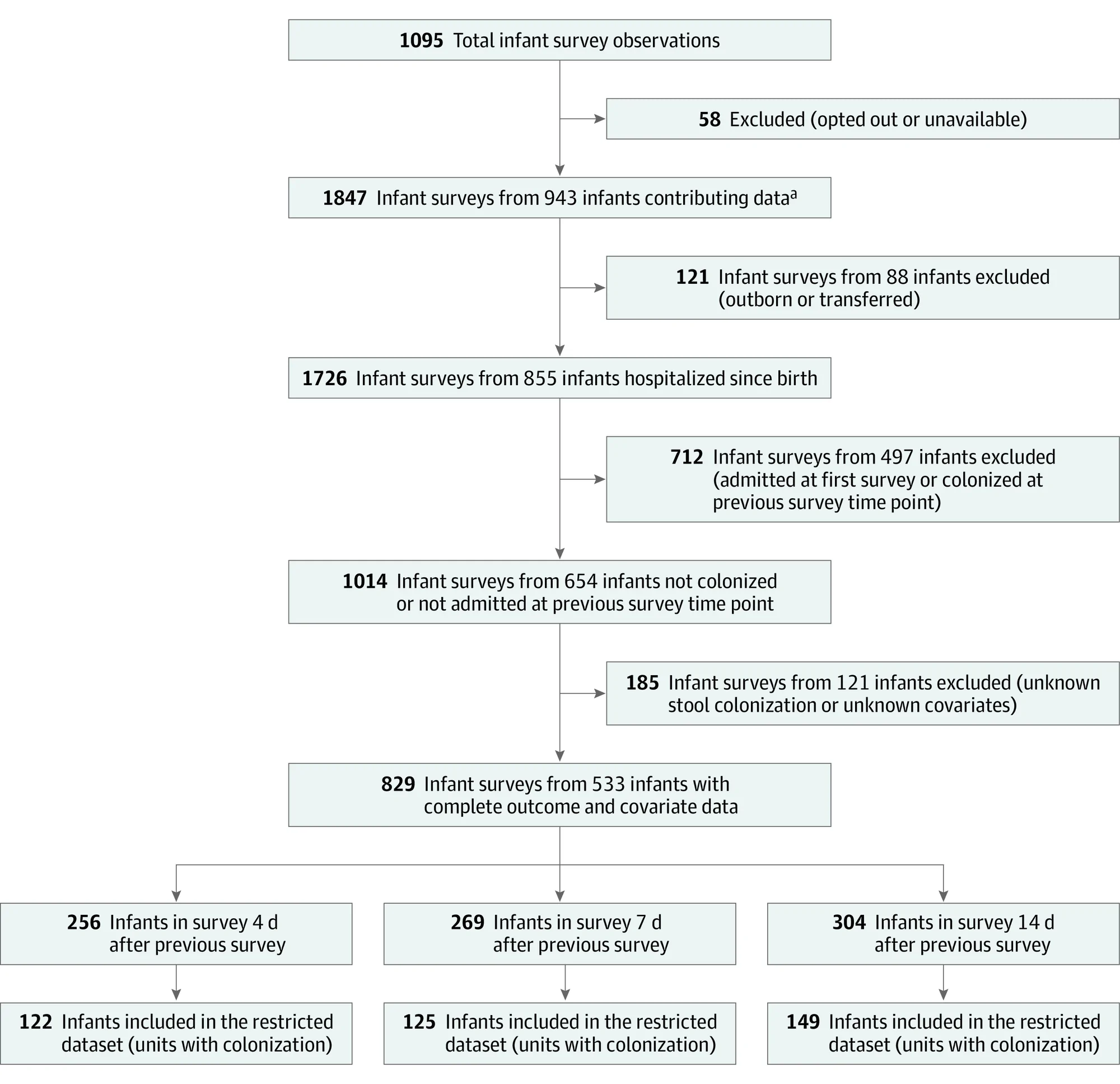
Flow Diagram Showing Infants in the Overall Study and Those Included in the Main Analysis and Sensitivity Analysis ^a^Infants contributing survey data include those that may have missing (not produced) stool samples.

A total of 533 infants contributing 829 complete infant surveys met inclusion criteria for the ESBL colonization acquisition analysis. Among these infants, median (IQR) gestational age at birth was 34 (30-37) weeks, median (IQR) birth weight was 2000 (1245-2990) g and median (IQR) length of stay at survey was 14 (5-32) days. Overall, 90 of 533 infants in the analysis population (16.9%) acquired ESBLs during follow-up (Figure 2).

There were 256 infants included in the 4-day interval dataset, 269 in the 7-day interval dataset, and 304 in the 14-day interval dataset (Table 2). A total of 33 of 256 (12.9%), 24 of 269 (8.9%), and 33 of 304 (10.9%) infants were ESBL-colonized in each interval dataset, respectively. Within interval-specific datasets, median (IQR [max]) prior colonization pressure was 0.06 (0.00-0.18 [0.71]) for the 4-day interval, 0.02 (0.00-0.18 [0.62]) for the 7-day interval and 0.05 (0.00-0.22 [0.67]) for the 14-day interval (eFigure 8 in Supplement 1).

**Table 2.** Characteristics of the Analysis Population Included in Each of the Main Models by Extended-Spectrum β-Lactamase (ESBL) Colonization Status^a^

Characteristic	Participants, No. (%)					
	Model 1: 4 d interval		Model 2: 7 d interval		Model 3: 14 d interval	
	Not ESBL colonized (n = 223)^a^	ESBL colonized (n = 33)^a^	Not ESBL colonized (n = 245)^a^	ESBL colonized (n = 24)^a^	Not ESBL colonized (n = 271)^a^	ESBL colonized (n = 33)^a^
Country						
Estonia	5 (2.2)	0	5 (2.0)	0	7 (2.6)	0
Germany	17 (7.6)	1 (3.0)	18 (7.3)	1 (4.2)	22 (8.1)	2 (6.1)
Greece	52 (23.3)	16 (48.5)	59 (24.1)	9 (37.5)	57 (21.0)	14 (42.4)
Italy	9 (4.0)	0	9 (3.7)	3 (12.5)	10 (3.7)	4 (12.1)
Poland	11 (4.9)	1 (3.0)	9 (3.7)	2 (8.3)	15 (5.5)	1 (3.0)
Spain	31 (13.9)	11 (33.3)	33 (13.5)	8 (33.3)	39 (14.4)	8 (24.2)
Switzerland	43 (19.3)	0	47 (19.2)	0	51 (18.8)	1 (3.0)
United Kingdom	55 (24.7)	4 (12.1)	65 (26.5)	1 (4.2)	70 (25.8)	3 (9.1)
Gestational age, median (IQR), wk	33.0 (28.0-37.0)	32.0 (31.0-37.0)	32.0 (28.0-36.0)	36.0 (30.0-38.0)	32.0 (28.0-36.0)	33.0 (31.0-37.0)
Gestational age group						
<28 wk	53 (23.8)	4 (12.1)	53 (21.6)	3 (12.5)	63 (23.2)	3 (9.1)
28 to <32 wk	46 (20.6)	6 (18.2)	52 (21.2)	5 (20.8)	64 (23.6)	6 (18.2)
32 to <37 wk	67 (30.0)	13 (39.4)	85 (34.7)	4 (16.7)	81 (29.9)	15 (45.5)
≥37 wk	57 (25.6)	10 (30.3)	55 (22.4)	12 (50.0)	63 (23.2)	9 (27.3)
Gestation						
<32 wk	99 (44.4)	10 (30.3)	105 (42.9)	8 (33.3)	127 (46.9)	9 (27.3)
≥32 wk	124 (55.6)	23 (69.7)	140 (57.1)	16 (66.7)	144 (53.1)	24 (72.7)
Birthweight, median (IQR), g	1805 (940-2705)	1830 (1430-2880)	1800 (1040-2580)	2255 (1560-3135)	1725 (995-2640)	1750 (1450-2640)
Postnatal age, median (IQR), d	20 (7-44)	17 (8-48)	15 (6-41)	13 (4-39)	15 (6-35)	12 (7-30)
Length of stay on NICU at survey, median (IQR), wk	2.6 (0.7-5.3)	1.6 (0.7-5.1)	1.86 (0.71-4.57)	1.14 (0.50-3.50)	1.9 (0.7-4.3)	1.3 (0.7-4.1)
Antibiotic exposure within 14 d	128 (57.4)	17 (51.5)	139 (56.7)	13 (54.2)	154 (56.8)	15 (45.5)
ESBL colonization pressure at previous survey, median (IQR)	0.04 (0.00-0.11)	0.18 (0.11-0.25)	0.00 (0.00-0.14)	0.25 (0.15-0.44)	0.05 (0.00-0.13)	0.22 (0.13-0.47)

^a^ Models included infant surveys who were not admitted yet or were uncolonized at the previous survey time point with known ESBL colonization status and known covariates. Previous surveys were 4 days, 7 days, or 14 days before.

**Figure 3.**
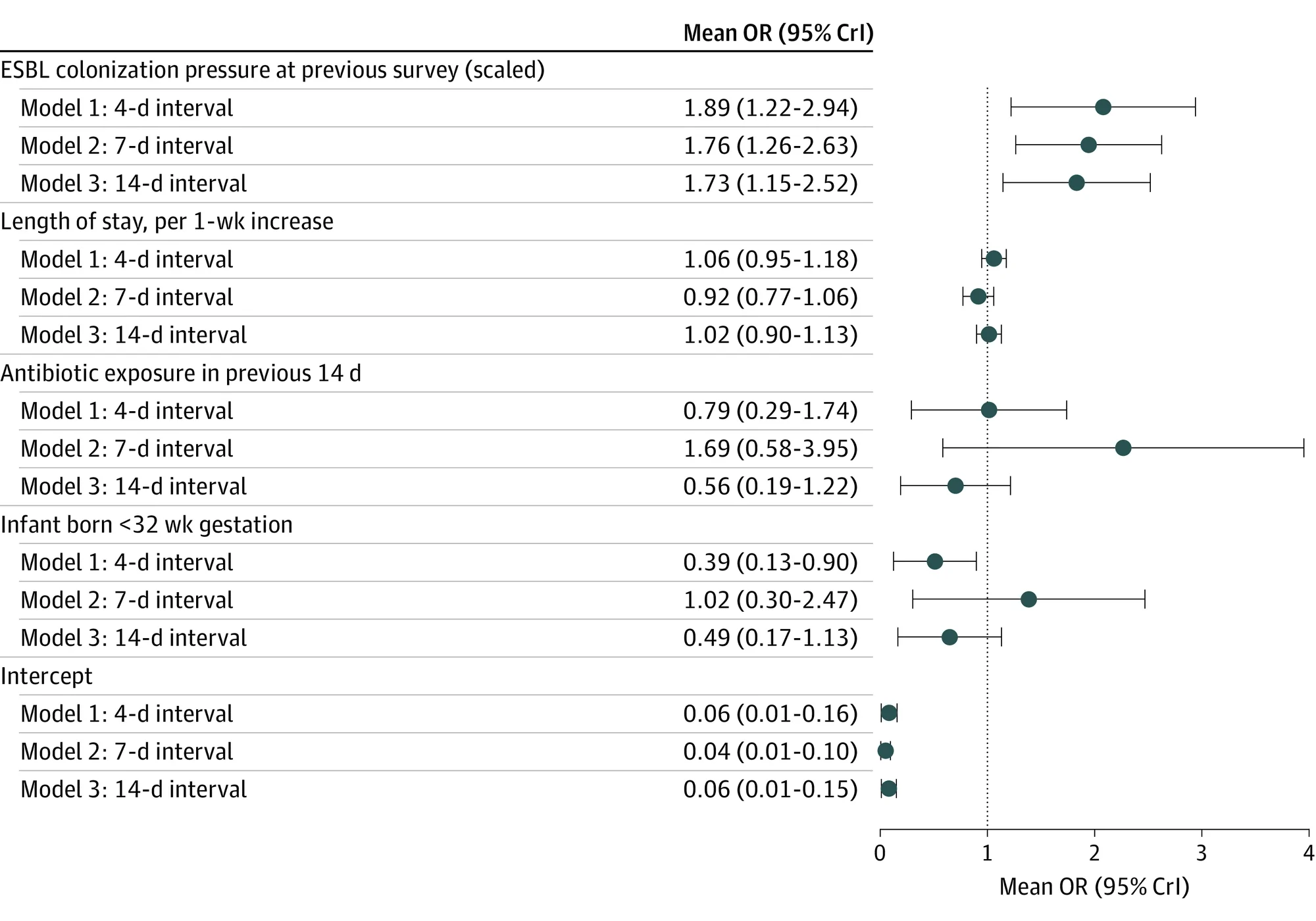
Mean Odds Ratio (OR) Estimates of Model Fixed Effects of 3 Primary Analysis Models Extended-spectrum β-lactamase (ESBL) colonization pressure at the previous survey was multiplied by 10 before the analysis so that coefficients represent the effect per 0.1 increase on the original proportional scale. Models were fit on 3 datasets where colonization pressure was measured from the previous survey (4 days, 7 days, and 14 days before, respectively). CrI indicates credible interval.

After adjusting for prematurity, LOS, prior antibiotic exposure, and neonatal unit, higher ESBL colonization pressure at the previous survey was consistently associated with increased odds of ESBL acquisition across all 3 models. The estimated mean ORs of ESBL acquisition per 10% increase in colonization pressure were 1.89 (95% CrI, 1.22-2.94) for the 4-day interval, 1.76 (95% CrI, 1.26-2.63) for the 7-day interval, and 1.73 (95% CrI, 1.15-2.52) for the 14-day interval (Figure 3 and eTable 9 in Supplement 1). There was substantial between-unit variability in baseline ESBL acquisition risk beyond that explained by measured covariates with standard deviation of the unit intercept (log-odds) of 1.51 (95% CrI, 0.74-2.60) for the 4-day interval, 0.83 (95% CrI, 0.05-1.89) for the 7-day model, and 1.32 (95% CrI, 0.45-2.43) in the 14-day model (eFigure 9 and eTable 10 in Supplement 1).

### Sensitivity Analysis

In our sensitivity analysis restricted to neonatal units with evidence of some ESBL colonization (eTable 11 in Supplement 1), point estimates for the association between colonization pressure and acquisition remained directionally consistent with the main models across all time intervals, although the credible intervals were wider and included the null value (eFigure 10 in Supplement 1). The largest point estimate was seen in the 4-day interval model (mean OR, 1.62; 95% CrI, 0.97-2.54). The estimate was smaller in the 14-day interval model (mean OR, 1.36; 95% CrI, 0.76-2.13), indicating greater uncertainty in effect size magnitude when colonization pressure was measured further from the outcome. Substantial between-unit variability in baseline ESBL acquisition risk remained (eFigure 10 and eTable 12 in Supplement 1).

## Discussion

In this multicenter study of European neonatal units, we found a consistent positive association between increasing ESBL colonization pressure and infant acquisition of ESBL gut colonization during hospitalization. Odds of ESBL acquisition increased 1.7 to 1.9 times for every 10-percentage point increase in colonization pressure on the neonatal unit. This association was observed across multiple exposure windows and persisted after adjustment for individual-level factors and unit-level clustering, highlighting colonization pressure as a potentially important unit-level risk factor for incident colonization by ESBL-harboring bacteria in the neonatal setting.

We observed ESBL colonization among both infants born at less than 32 and 32 or more weeks’ gestation for many surveys. While infants of higher gestational age are generally at lower risk of developing invasive neonatal sepsis,^25,26^ the presence of colonization in both groups suggests that resistant bacterial colonization is not confined to infants at high risk of infection,^27^ with all hospitalized infants potentially contributing to colonization pressure on the neonatal unit. This supports the importance of unit-wide IPC approaches in addition to strategies that focus on specific high-risk subgroups.^28^ Strategies that decrease resistant bacterial colonization in individual infants may confer direct benefits to those infants while also providing indirect benefits by reducing the overall colonization pressure on the unit.

The observed association between increasing colonization pressure and acquisition risk likely reflects the cumulative effect of acquisition and transmission opportunities within the neonatal unit environment and may also capture unmeasured differences in IPC practices between units. Neonatal units are complex, shared-care environments creating conditions that facilitate cross-transmission.^19^ Colonization pressure represents a measurable, unit-level variable that could be used as a summary indicator of acquisition risk which could be useful for decisions about IPC escalation during outbreaks or high colonization pressure periods, but further investigation is needed.

We found high between-unit variation in baseline colonization acquisition risk which suggests local factors also play a role in acquisition risk. This may reflect different implementation levels of IPC measures, surveillance, and antibiotic policies, which were captured as part of the unit-level random effect but were not measured directly during the study. Skin-to-skin contact^29,30^ and milk feeds^31^ could also have important IPC effects and could differ in their implementation between units. More research on the impact of specific structural factors, such as staffing^10,18^ and layout and implementation levels of specific IPC measures^32,33^ are warranted to untangle their influence on colonization acquisition risk. Future clinical trials could investigate which interventions reduce colonization pressure and whether they in turn reduce acquisition.

Our study design fills gaps from prior neonatal colonization studies. Previous investigations have focused on individual-level risk factors within single centers or outbreak settings, examined combined colonization and infection outcomes and have not explicitly assessed unit-level risk such as colonization pressure.^11,31,34,35,36,37^ Two studies, 1 in Europe and 1 in the US, showed a small increase in odds (ORs, 1.05 and 1.06, respectively) of MRSA acquisition with increasing colonization pressure.^12,20^ By conducting repeated, unit-wide cross-sectional surveys in a multicenter, multinational study and sampling all infants present at predefined time points, we were able to assess colonization pressure associated with acquisition of ESBLs while adjusting for other individual risk-factors. Neonatal literature on colonization pressure and resistant bacteria acquisition of Gram-negative bacteria is limited and largely from low and middle-income settings. Studies show higher numbers of ESBL-colonized infants increase acquisition risk in Kenyan units,^8^ are associated with indirect transmissions in Madagascan units,^19^ and that colonized infants are the primary source of ESBL *K. pneumoniae* in a Cambodian unit.^18^ Our findings align with these other studies and, importantly, explicitly show this for ESBL-harboring bacteria in a multicountry European neonatal setting. Our findings also align with evidence from adult intensive care settings in Europe, where colonization pressure has been shown to be a key driver of transmission of resistant bacteria in hospital settings.^13,14,15,16,38,39^

### Limitations and Strengths

We used surveillance methods that are feasible, scalable, and affordable for routine use, including cross-sectional sampling with limited clinical data collection and PCR-based detection of priority resistance genes without searching for specific bacteria carrying these ARGs. This approach allowed inclusion of large numbers of infants across settings but did not permit identification of bacterial species, knowledge of ARG expression, reconstruction of transmission pathways, or determining the relative contribution of different infant groups to transmission dynamics, which would require whole-genome sequencing.^18^ While we focused our analysis on acquisition of ESBL genes due to low prevalence of other genes in most of these units, further investigation is needed to understand the relationship between colonization pressure and other resistance genes with lower prevalence and between colonization pressure and acquisition of wild-type susceptible organisms.

This study has several other limitations. It was observational and cross-sectional, and missing stool samples may have led to underestimation of colonization pressure or acquisition. We assumed inborn infants admitted between survey time points were not colonized on admission which may have resulted in selection bias and overestimated the number of infants that had hospital acquired colonization; however, there was no significant difference in colonization status by inclusion criteria (eTable 9 in Supplement 1). While we adjusted for key identified confounders (eFigure 3 in Supplement 1), residual confounding of unmeasured or imprecisely measured factors remains possible. We did not include breast milk exposure or skin-to-skin contact due to the complexity of their individual and unit-level relationships and because our study design was not structured to measure their implementation levels. Antibiotic exposure was included as a binary exposure with potential limited granularity and potential insufficient adjustment for the associations with specific antibiotics. Importantly, our confounder selection was focused on estimating the association between colonization pressure and acquisition. We did not aim to evaluate the independent causal effects of individual-level factors and purposefully excluded factors we considered to be specific risk factors for infection.

Despite these limitations, this study represents one of the largest multicenter, multinational assessments of resistant bacterial colonization and colonization pressure in neonatal units to date. We demonstrate a robust association between ward-level ESBL colonization pressure and subsequent ESBL acquisition, independent of prematurity, length of stay, and antimicrobial exposure across 24 tertiary neonatal units in Europe with high variation in baseline colonization risk. This positive association persisted, although with greater uncertainty, in our sensitivity analyses focusing only on units with some baseline ESBL colonization.

## Conclusions

In this cross-sectional study of hospitalized neonates in Europe, we found an approximately 73% to 89% increased odds of ESBL acquisition per 10–percentage point increase in unit-level ESBL colonization prevalence over a period of 4 to 14 days. Colonization occurred across gestational age groups, highlighting the importance of unit-wide approaches to IPC. Interventions that reduce colonization, acting both directly and indirectly, may be particularly important in units with high colonization pressure and during outbreaks, and should be considered alongside strategies focused on preventing invasive infection in the most vulnerable infants.

## Notes

Supplement 1. eMethods. eTable 1. Site Characteristics of Participating Neonatal Units eTable 2. Overview of Antibiotic Resistance Genes/Bacteria Analyzed and Kits Used for Analysis eTable 3. Overview of Methicillin Resistant Organisms Detected Based on Detected Gene Targets in the DiarellaMRSA seqC Real-Time PCR Kit eFigure 1. Illustration of the Schema for Constructing Analysis Datasets for Different Example Sites Where the Order of Intervals Was Different eFigure 2. Example Illustrations of How Infants Present on the Unit Across Multiple Surveys Contribute to the Colonization Pressure Exposure and the Incident Colonization Outcome eFigure 3. Directed Acyclic Graph (DAG) for Relationship Between Potential Confounders and Colonization Pressure and Infant ESBL Gut Colonization eTable 4. Priors Trialed for Model Fitting eFigure 4. Comparison of Prior Distribution and Posterior Distribution of Covariate and SD Using Default Priors eFigure 5. Comparison of Posterior Distribution for Covariates and Between Site (Neonatal Unit) SD When Using the 3 Different Trialed Priors for the Main Analysis Population eFigure 6. Posterior Predictive Checks of the Main 3 Models (4-, 7-, and 14-Day Intervals) Showing Posterior Predictive Draws of ESBL Colonization and the Observed Mean Probability of ESBL Coloniszation by Neonatal Unit eTable 5. Table of Overall Study Population Including all Infants Surveyed by Survey Number eTable 6. Stool Resistant Bacterial Colonization Patterns Detected in the Study eTable 7. Antibiotic Resistance Genes Detected in Available Stool Collected Samples by Survey Number eTable 8. Antibiotic Resistance Gene Targets Detected in Collected Stool Samples and Skin Swabs by Neonatal Unit eFigure 7. Number of Infants by ESBL Colonization Status and Prematurity Status eFigure 8. Colonization Pressure (Proportion of Infants That Were ESBL-Colonized at the Previous Survey Time Point) for Each Model Fitting Dataset Where the Previous Survey Time Point Occurred 4 Days, 7 Days or 14 Days Before the Analyzed Survey eTable 9. Table Showing Eligibility Status for Incident Acquisition – Either Not Colonized at Previous Survey or Newly Admitted Since Previous Survey – and Colonization Status by Analysis Dataset eTable 10. Model Coefficients (Mean Odds of Posterior Draws), 95% Credible Interval and Probability of Direction of Association for the 3 Main Analysis Models Fit Using Colonization Pressure at the Previous Survey With Intervals Between Surveys of 4 Days, 7 Days, and 14 Days eFigure 9. Neonatal Unit Random Effects Odds Ratio Deviations From the Global Intercept (Mean and 95% Credible Interval) of 3 Models eTable 11. Characteristics of Infants Included in the Sensitivity Analysis Restricted to Neonatal Units With Colonization Pressure >0 at all 4 Surveys for Each Model-Fitting Dataset for 4-Day Interval, 7-Day Interval, and 14-Day Interval eFigure 10. Odds Ratio Estimates (Mean and 95% Credible Interval) of Model Fixed Effects of 3 Sensitivity Analysis Models eTable 12. Model Coefficients (Mean Odds of Posterior Draws), 95% Credible Interval and Probability of Direction of Association for the 3 Sensitivity Analysis Models Fit Using Colonization Pressure at the Previous Survey With Intervals Between Surveys of 4 Days, 7 Days, and 14 Days eReferences.

Supplement 2. Data Sharing Statement
