# Supplementary material for: Neonatal Unit Colonization Pressure and Acquisition of Extended-Spectrum β-Lactamase Gut Colonization
Source: JAMA Netw Open. 2026 Sep 15;9(9):e2632644. doi: 10.1001/jamanetworkopen.2026.32644 (PMC13579436; doi:10.1001/jamanetworkopen.2026.32644)
Supplement: Supplementary file 1 [file jamanetwopen-e2632644-s001.pdf]

Supplemental Online Content

Cook A, Berkell M, van Werkhoven H, et al. The association between neonatal unit colonization pressure and acquisition of extended-spectrum β-lactamase gut colonization. *JAMA Netw Open*. 2026;9(9):e2632644. doi:10.1001/jamanetworkopen.2026.32644

eMethods.

eTable 1. Site Characteristics of Participating Neonatal Units

eTable 2. Overview of Antibiotic Resistance Genes/Bacteria Analyzed and Kits Used for Analysis

eTable 3. Overview of Methicillin Resistant Organisms Detected Based on Detected Gene Targets in the DiarellaMRSA seqC Real-Time PCR Kit

eFigure 1. Illustration of the Schema for Constructing Analysis Datasets for Different Example Sites Where the Order of Intervals Was Different

eFigure 2. Example Illustrations of How Infants Present on the Unit Across Multiple Surveys Contribute to the Colonization Pressure Exposure and the Incident Colonization Outcome

eFigure 3. Directed Acyclic Graph (DAG) for Relationship Between Potential Confounders and Colonization Pressure and Infant ESBL Gut Colonization

eTable 4. Priors Trialed for Model Fitting

eFigure 4. Comparison of Prior Distribution and Posterior Distribution of Covariate and SD Using Default Priors

eFigure 5. Comparison of Posterior Distribution for Covariates and Between Site (Neonatal Unit) SD When Using the 3 Different Trialed Priors for the Main Analysis Population

eFigure 6. Posterior Predictive Checks of the Main 3 Models (4-, 7-, and 14-Day Intervals) Showing Posterior Predictive Draws of ESBL Colonization and the Observed Mean Probability of ESBL Coloniszation by Neonatal Unit

eTable 5. Table of Overall Study Population Including all Infants Surveyed by Survey Number

eTable 6. Stool Resistant Bacterial Colonization Patterns Detected in the Study

eTable 7. Antibiotic Resistance Genes Detected in Available Stool Collected Samples by Survey Number

eTable 8. Antibiotic Resistance Gene Targets Detected in Collected Stool Samples and Skin Swabs by Neonatal Unit

eFigure 7. Number of Infants by ESBL Colonization Status and Prematurity Status

© 2026 Cook A et al. *JAMA Network Open*.

eFigure 8. Colonization Pressure (Proportion of Infants That Were ESBL-Colonized at the Previous Survey Time Point) for Each Model Fitting Dataset Where the Previous Survey Time Point Occurred 4 Days, 7 Days or 14 Days Before the Analyzed Survey

eTable 9. Table Showing Eligibility Status for Incident Acquisition – Either Not Colonized at Previous Survey or Newly Admitted Since Previous Survey – and Colonization Status by Analysis Dataset

eTable 10. Model Coefficients (Mean Odds of Posterior Draws), 95% Credible Interval and Probability of Direction of Association for the 3 Main Analysis Models Fit Using Colonization Pressure at the Previous Survey With Intervals Between Surveys of 4 Days, 7 Days, and 14 Days

eFigure 9. Neonatal Unit Random Effects Odds Ratio Deviations From the Global Intercept (Mean and 95% Credible Interval) of 3 Models

eTable 11. Characteristics of Infants Included in the Sensitivity Analysis Restricted to Neonatal Units With Colonization Pressure >0 at all 4 Surveys for Each Model-Fitting Dataset for 4-Day Interval, 7-Day Interval, and 14-Day Interval

eFigure 10. Odds Ratio Estimates (Mean and 95% Credible Interval) of Model Fixed Effects of 3 Sensitivity Analysis Models

eTable 12. Model Coefficients (Mean Odds of Posterior Draws), 95% Credible Interval and Probability of Direction of Association for the 3 Sensitivity Analysis Models Fit Using Colonization Pressure at the Previous Survey With Intervals Between Surveys of 4 Days, 7 Days, and 14 Days

eReferences.

This supplemental material has been provided by the authors to give readers additional information about their work.

© 2026 Cook A et al. *JAMA Network Open*.

eMethods.

Neonatal units

A summary of the neonatal units selected for the colonisation assessment are in eTable 1. Sites were selected as potential clinical trial sites for NeoDeco and units eligible for inclusion were those providing routine care of extremely premature babies (e.g. those <28 weeks gestation).

eTable 1. Site Characteristics of Participating Neonatal Units.

Site	Country	Hospital type	Obstetric unit directly linked	Postnatal ward directly linked	Highest level of care available	Neonatal surgery performed	Total number of neonatal cots at full capacity	Admissions per year[1] (total)	% admissions born <32 weeks' gestation
CH2	Switzerland	Tertiary/teaching hospital	Yes	Yes	Neonatal Intensive Care Unit	Yes	23	724	8.3%
CH1	Switzerland	Standalone paediatric hospital	Yes	No	Neonatal Intensive Care Unit	Yes	30	529	14.6%
CH3	Switzerland	Tertiary/teaching hospital	Yes	Yes	Neonatal Intensive Care Unit	Yes	40	835	12.7%
CH4	Switzerland	Tertiary/teaching hospital	Yes	Yes	Neonatal Intensive Care Unit	Yes	38	450	17.8%
DE1	Germany	Tertiary/teaching hospital	Yes	Yes	Neonatal Intensive Care Unit	Yes	50	1,300	7.3%
ES1	Estonia	Standalone paediatric hospital	No	No	Neonatal Intensive Care Unit	Yes	16	113	26.5%
ES2	Estonia	Tertiary/teaching hospital	Yes	Yes	Neonatal Intensive Care Unit	Yes	9	167	18.0%
GR2	Greece	Tertiary/teaching hospital	Yes	Yes	Neonatal Intensive Care Unit	No	16	308	9.4%
GR1	Greece	Tertiary/teaching hospital	No	No	Neonatal Intensive Care Unit	Yes	27	233	18.0%
GR3	Greece	Tertiary/teaching hospital	Yes	Yes	Neonatal Intensive Care Unit	Yes	40	464	44.8%
GR4	Greece	Tertiary/teaching hospital	Yes	Yes	Neonatal Intensive Care Unit	Yes	25	541	9.8%
GR6	Greece	Tertiary/teaching hospital	Yes	Yes	Neonatal Intensive Care Unit	No	25	286	9.1%
GR5	Greece	Tertiary/teaching hospital	Yes	Yes	Neonatal Intensive Care Unit	Yes	28	615	13.5%
IT1	Italy	Tertiary/teaching hospital	Yes	Yes	Neonatal Intensive Care Unit	Yes	29	450	16.0%
IT2	Italy	Tertiary/teaching hospital	Yes	Yes	Neonatal Intensive Care Unit	Yes	12	991	0.8%
PL1	Poland	Tertiary/teaching hospital	No	No	Neonatal Intensive Care Unit	Yes	50	1,100	9.1%
SP1	Spain	Tertiary/teaching hospital	Yes	Yes	Neonatal Intensive Care Unit	Yes	36	651	6.0%
SP2	Spain	Tertiary/teaching hospital	Yes	Yes	Neonatal Intensive Care Unit	Yes	38	147	26.5%
SP4	Spain	Tertiary/teaching hospital	Yes	Yes	Neonatal Intensive Care Unit	Yes	19	322	4.3%
SP3	Spain	Tertiary/teaching hospital	Yes	Yes	Neonatal Intensive Care Unit	Yes	41	500	21.8%
UK1	UK	Tertiary/teaching hospital	Yes	Yes	Neonatal Intensive Care Unit	No	31	566	19.1%
UK3	UK	Tertiary/teaching hospital	Yes	Yes	Neonatal Intensive Care Unit	Yes	40	543	28.9%
UK4	UK	Tertiary/teaching hospital	Yes	Yes	Neonatal Intensive Care Unit	Yes	69	1,073	17.7%
UK2	UK	Tertiary/teaching hospital	Yes	Yes	Neonatal Intensive Care Unit	Yes	34	978	11.2%

[1]Admissions were for the most recent year available when units completed this information at the start of the survey period (2020 for 1 unit, 2021 for 16 units, 2022 for 5 units, 2023 for 2 units).

© 2026 Cook A et al. *JAMA Network Open*.

Sample collection

Skin swab samples were collected using Regular Flocked Swabs. For each skin swab sample, two swabs were placed into one collection tube with in-house bacterial storage medium. Each swab was moistened with sterile saline before sampling. The first swab was applied between two cervical skin folds (2 swabs, 180 degrees around the neck). The umbilicus was sampled with a second swab by swiping it 360 degrees two times around the base of the umbilicus while rotating the tip without touching the umbilicus itself.

Stool samples were collected into faecal containers with a screw cap and spoon by, when changing the nappies of infants, collecting as much faecal matter as possible using the spoon and sealing the container.

Skin swab collection tubes and stool containers containing sample material were all stored at 4°C immediately after collection until they could be stored at <-70°C at the local site. All collected samples in this study were then shipped for biobanking at -80°C and analysis at the Laboratory of Medical Microbiology, University of Antwerp, Belgium.

PCR-based antimicrobial resistance detection

DNA was extracted from all samples using NucliSENS easyMAG (bioMérieux, France). Selected assays for stool and skin samples are described ineTable 2. A sample was considered positive for the relevant gene when a Ct <37 was detected for selected assays.

eTable 2. Overview of Antibiotic Resistance Genes/Bacteria Analyzed and Kits Used for Analysis.

**Gram stain**	**Type of resistance and gene targets**	**Selected chromogenic medium**	**Selected RT-qPCR assay**	**Sample type analysed**
Gram-negative bacteria	Carbapenemase-producing Enterobacterales (CPEs): bla_{KPC}, bla_{VIM}, bla_{NDM}, $bla_{OXA\text{-}48}$, bla_{IMP}	ChromID™ CARBA agar (Biomérieux) ChromID® OXA-48 (Biomérieux)	Carbaplex IVD PCR kit (Bruker, Part No. 1849975), 100 tests	Stool
	Beta-lactamase-producing *Enterobacterales* (ESBLs): $bla_{CTX\text{-}M}$	ChromID ESBL (Biomérieux)	blaCTX-M Ba04646149_s1-FAM (group 1) and Ba04646127_s1-VIC (group 9), Thermo Fisher	Stool
	beta-lactamase-producing *Enterobacterales* (ESBLs): bla_{SHV} **Note:* SHV positive samples not considered ESBL without further testing	ChromID ESBL (Biomérieux)	blaSHV Ba04646134_s1-FAM/MGB-NFQ (ThermoFisher) (cat#50133, S:360rxns)	Stool
Gram-positive bacteria	Vancomycin resistance: *vanA, vanB*	Enterococcosel agar with vancomycin (VRE selective) (BD, in-house preparation)	Geneproof VRE PCR kit (VRE/ISEX/25 rxn or 100rxn)	Stool
	Methicillin resistance: *mecA/mecC, SCCmec/orfX*	CHROMagar MRSA (Biotrading, in-house preparation)	DiarellaMRSA seqC real-time PCR kit (Gerbion)	Skin swab

© 2026 Cook A et al. *JAMA Network Open*.

Extracted DNA from skin swab samples was further analysed with DiarellaMRSA seqC real-time PCR kit for detection of *mecA/mecC and SCCmec/orfX*. See eTable 3 for assessment of methicillin resistant organisms based on gene targets detected.

eTable 3. Overview of Methicillin Resistant Organisms Detected Based on Detected Gene Targets in the DiarellaMRSA seqC Real-Time PCR Kit.

Gene targets detected	Resistant organism
mecA/mecC, SCCmec/orfX	MRSA
mecA/mecC,	MRCoNS

Culture-based antimicrobial resistance detection

As a quality control and comparator assay, 25% of samples with sufficient material were taken forward for analysis by quantitative culture.

Stool samples selected for analysis with quantitative culture of sufficient quantity (≥700 mg) were selected for screening for presence of ESBL, CPE, and VRE by quantitative culture (n = 359, 359/1448, 24.8%). 500 mg stool was liquefied and homogenised for quantitative culture on ChromID™ CARBA agar, ChromID® OXA-48, ChromID ESBL, and Enterococcosel agar with vancomycin, as well as blood agar (positive control) (eTable 2).

Skin swab samples (n = 233, 233/1830, 12.7%) were thawed, vortexed, and screened for presence of MRSA by quantitative culture by plating on CHROMagar MRSA and blood agar (positive control) (eTable 2).

Model fitting and prior sensitivity checks

We fit models separately for the datasets using the different intervals for colonisation pressure at the previous survey of 4 days, 7 days or 14 days. An illustration of this is for example sites is in eFigure 1. Our outcome of interest was detection of ESBL genes in stool sample and primary exposure of interest was unit-level colonisation pressure at the previous survey time point. The incident colonisation outcome was restricted to infants born at the participating site and hospitalised from birth. Incident colonisation only measures infants newly colonised at that survey time point - they must have been negative at the previous survey or newly admitted to the unit between surveys. Colonisation pressure is measured using all infants present on that survey with known colonisation status. An infant can contribute to colonisation pressure measurement multiple times if they remain on the unit and are colonised at multiple surveys. Examples of how infants contribute to the exposure and outcome are illustrated in eFigure 2.

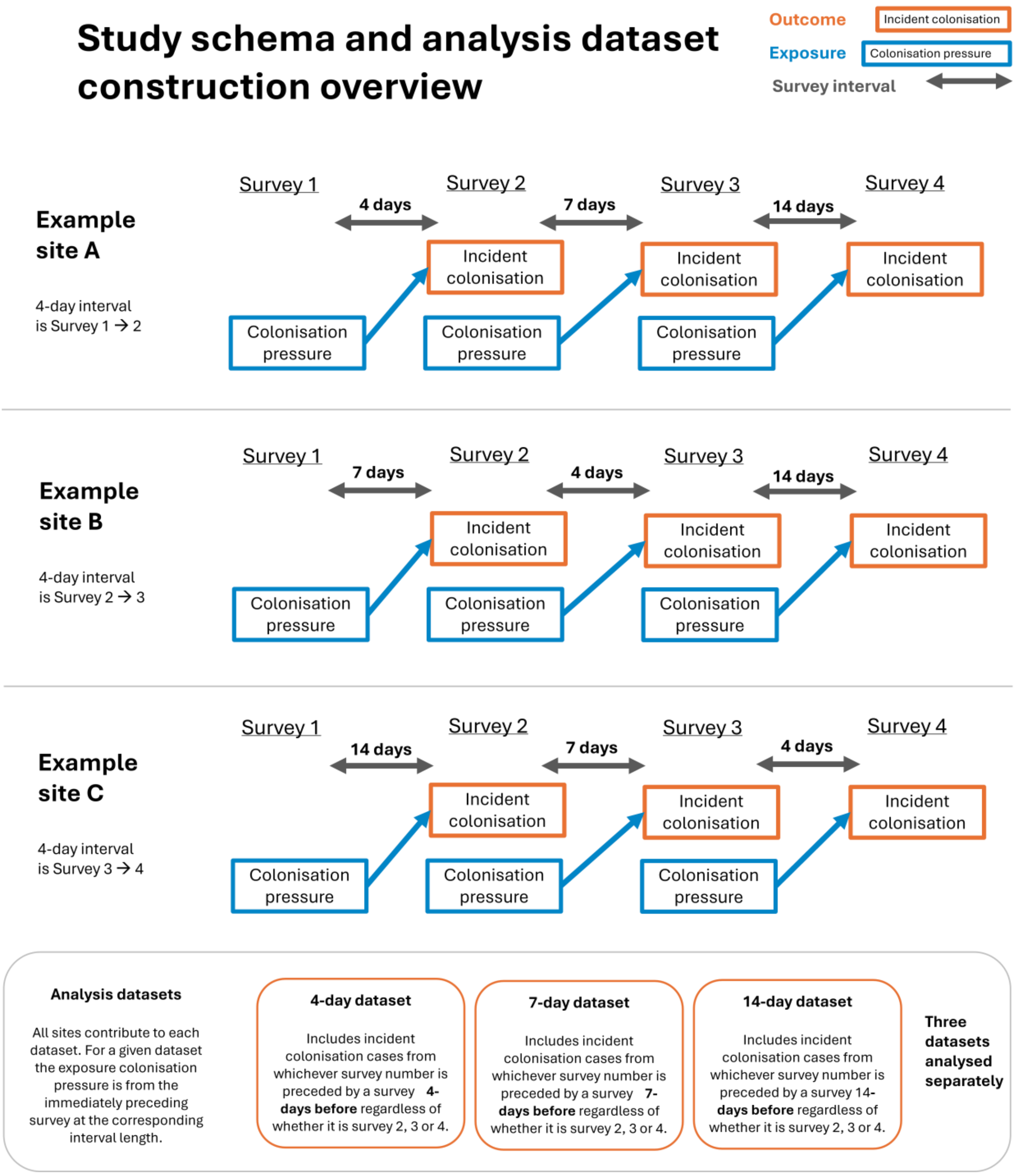

eFigure 1. Illustration of the Schema for Constructing Analysis Datasets for Different Example Sites Where the Order of Intervals Was Different

© 2026 Cook A et al. *JAMA Network Open*.

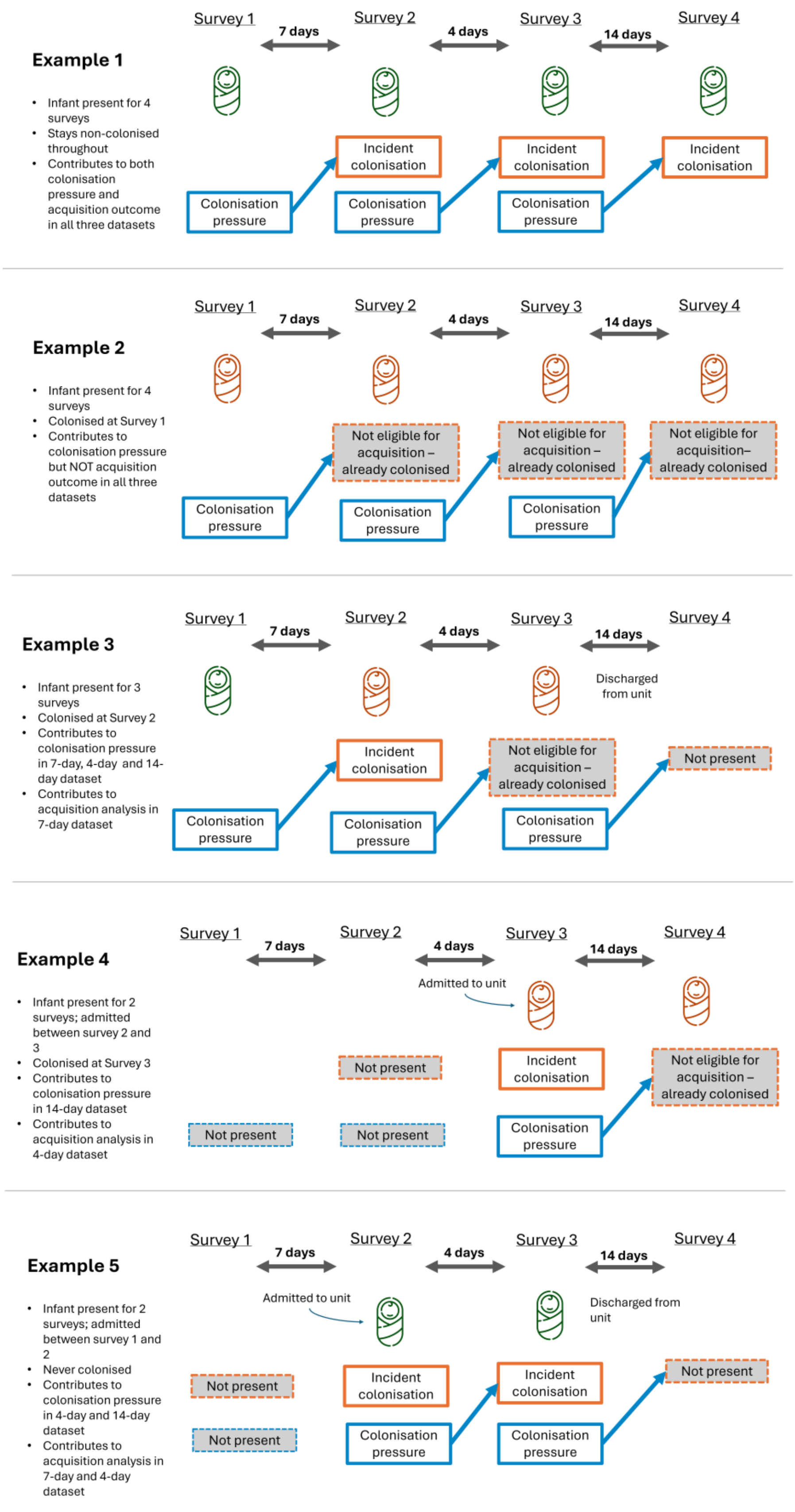

eFigure 2. Example Illustrations of How Infants Present on the Unit Across Multiple Surveys Contribute to the Colonisation Pressure Exposure and the Incident Colonisation Outcome. Infants eligible for the incident acquisition outcome were those born at the participating hospital and hospitalised since birth.

© 2026 Cook A et al. *JAMA Network Open*.

Confounder selection

Our analysis focused on estimating the association between unit-level colonisation pressure and infant-level acquisition of gut colonisation during neonatal unit admission. We adjusted for variables considered *a priori* to be potential confounders — defined as variables that may causally influence both colonisation pressure and infant colonisation acquisition but are not on the causal pathway between them. Confounders were selected based on clinical knowledge and biological plausibility and are illustrated in the directed acyclic graph (DAG) shown in eFigure 3.

Based on this framework, we adjusted for length of stay on the neonatal unit (weeks), antibiotic exposure in the preceding 14 days, and prematurity status (<32 weeks vs ≥32 weeks gestation). We use prematurity status of these cutoffs as within the wider NeoIPC project and NeoDeco trial, these are the cutoffs used for the population of interest.

We did not adjust for variables such as central line use, total parenteral nutrition (TPN), surgery, or mode of delivery. In the DAG, these variables were conceptualised as downstream consequences of baseline clinical characteristics (e.g. prematurity) or as potential mediators in the broader pathway linking colonisation to subsequent infection. Conditioning on such variables could result in over-adjustment or collider bias and would not be appropriate when estimating the total effect of colonisation pressure on colonisation acquisition. Instead, we aimed to clearly separate determinants of colonisation acquisition from determinants of infection risk.

At the neonatal unit level, we did not explicitly adjust for factors such as antibiotic stewardship policies, infection prevention and control (IPC) practices, hand hygiene compliance, or surveillance intensity. The impact of these factors on colonisation pressure and colonisation acquisition depends on their effectiveness and implementation fidelity, which were not directly measured in this study. Instead, we included a neonatal unit random effect to account for unmeasured unit-level heterogeneity.

We assumed that the inborn infants admitted between survey timepoints were not colonised at admission, consistent with evidence from low-and middle-income settings suggesting low rates of neonatal colonisation on admission to NICUs, despite high maternal colonisation prevalence in these settings[2-4], and lower documented maternal colonisation in European settings.[5,6] See eResults eTable 9. Tablefor a comparison of colonisation status by acquisition analysis eligibility (negative at previous survey or newly admitted).

Although infant breastmilk receipt and skin-to-skin contact (kangaroo care) may influence gut colonisation and infection risk[7], these factors may also affect microbial transmission dynamics at the unit level. Including these variables at both the individual and aggregate unit level could introduce circularity, as individual infants contribute to overall unit-level exposure patterns. For this reason, these variables were not included in the primary adjustment set, but their role is being explored in the ongoing NeoDeco cluster randomised trial within the NeoIPC programme (https://clinicaltrials.gov/study/NCT05993442).

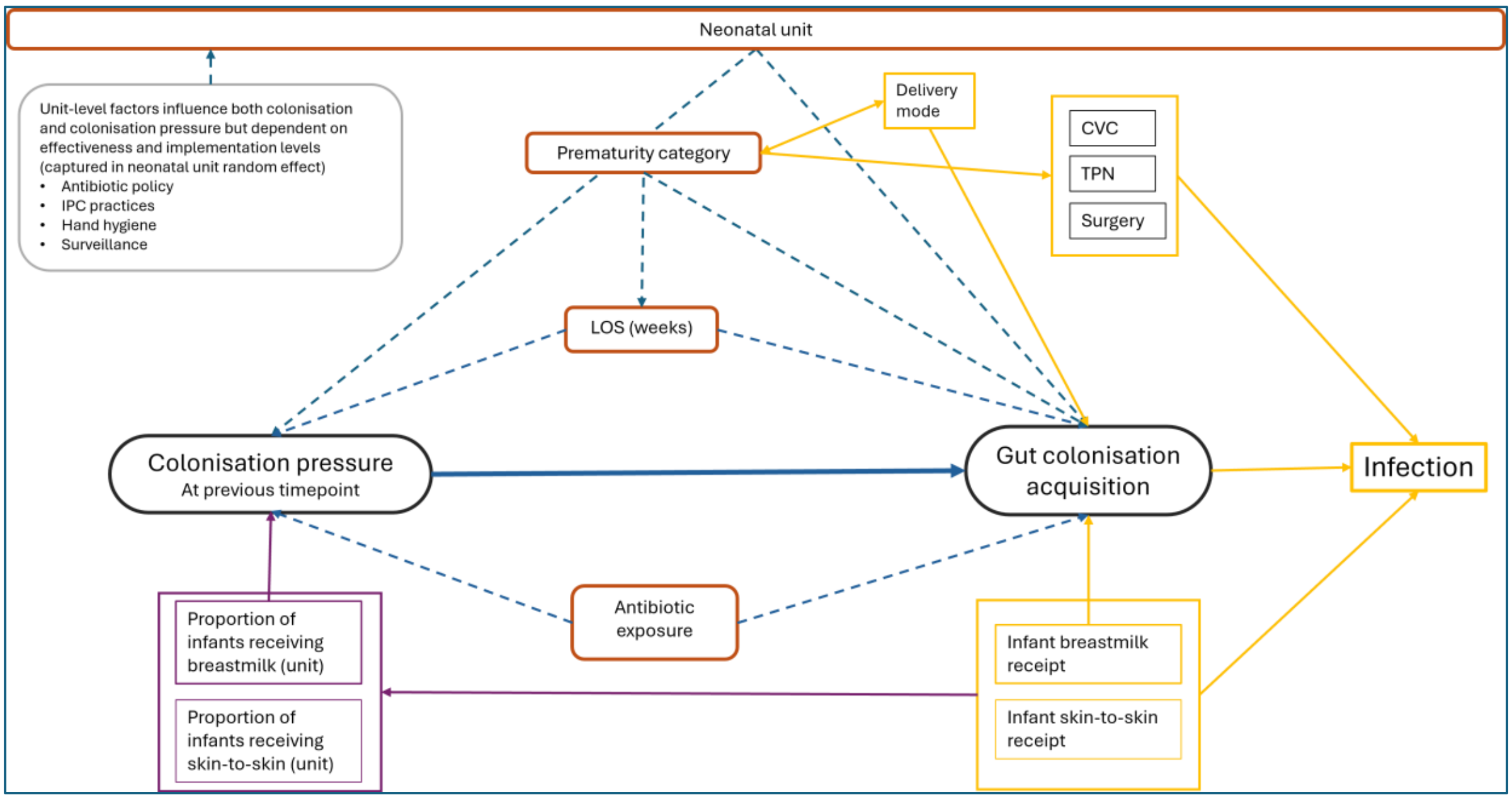

eFigure 3. Directed Acyclic Graph (DAG) for Relationship Between Potential Confounders and Colonization Pressure and Infant ESBL Gut Colonization. Primary exposure is in blue and outcome of interest is in red and confounders included in the model are in orange. Other variables considered as downstream or colliders or not confounders on the colonisation pressure → gut colonisation pathway are in yellow and purple. Dashed and solid blue arrows illustrate the relationships between variables we consider our confounders to have in the model and solid arrows in yellow and purple illustrate the relationships between variables not included in the model.

Prior sensitivity checks

For each dataset, we trialled models with different sets of priors on the coefficients, intercept and standard deviation. We conducted prior predictive checks to check for plausible effect sizes before observing the data. We assessed the priors and posterior distributions for the included parameters and across the different sets of priors to assess sensitivity of the model to prior specification.

Priors trialled are in eTable 4 and eFigure 4 shows the default priors selected against the posteriors for the covariates for each model. eFigure 5 shows the posterior distribution for each covariate from models fit using the different priors.

eTable 4. Priors Trialed for Model Fitting.

	Coefficient	Intercept	Between unit SD
Default	Normal(0, 1.5)	Student t(3, -2, 1.5)	Exponential(1)
Tight	Normal(0, 1.0)	Student t(3, -2, 1.5)	Exponential(2)
Wide	Normal(0, 2.5)	Student t(3, -2, 1.5)	Student t(3, 0, 2.5)*

**positive-restricted from the T-distribution*

© 2026 Cook A et al. *JAMA Network Open*.

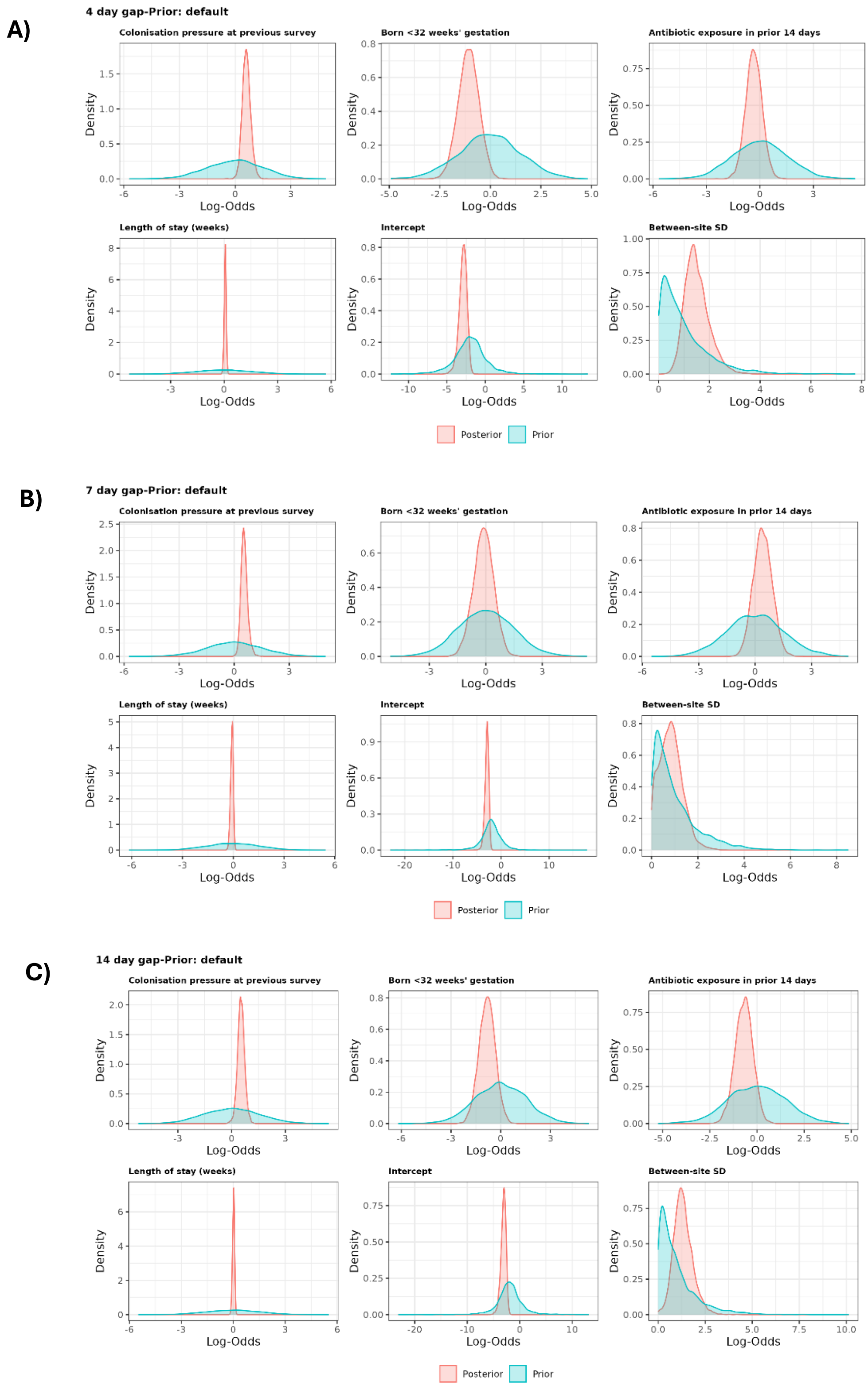

eFigure 4. Comparison of Prior Distribution and Posterior Distribution of Covariate and SD Using Default Priors for A) model fitting dataset using colonisation pressure at survey 4 days prior, B) colonisation pressure at survey 7 days prior and C) colonisation pressure at survey 14 days prior.

© 2026 Cook A et al. *JAMA Network Open*.

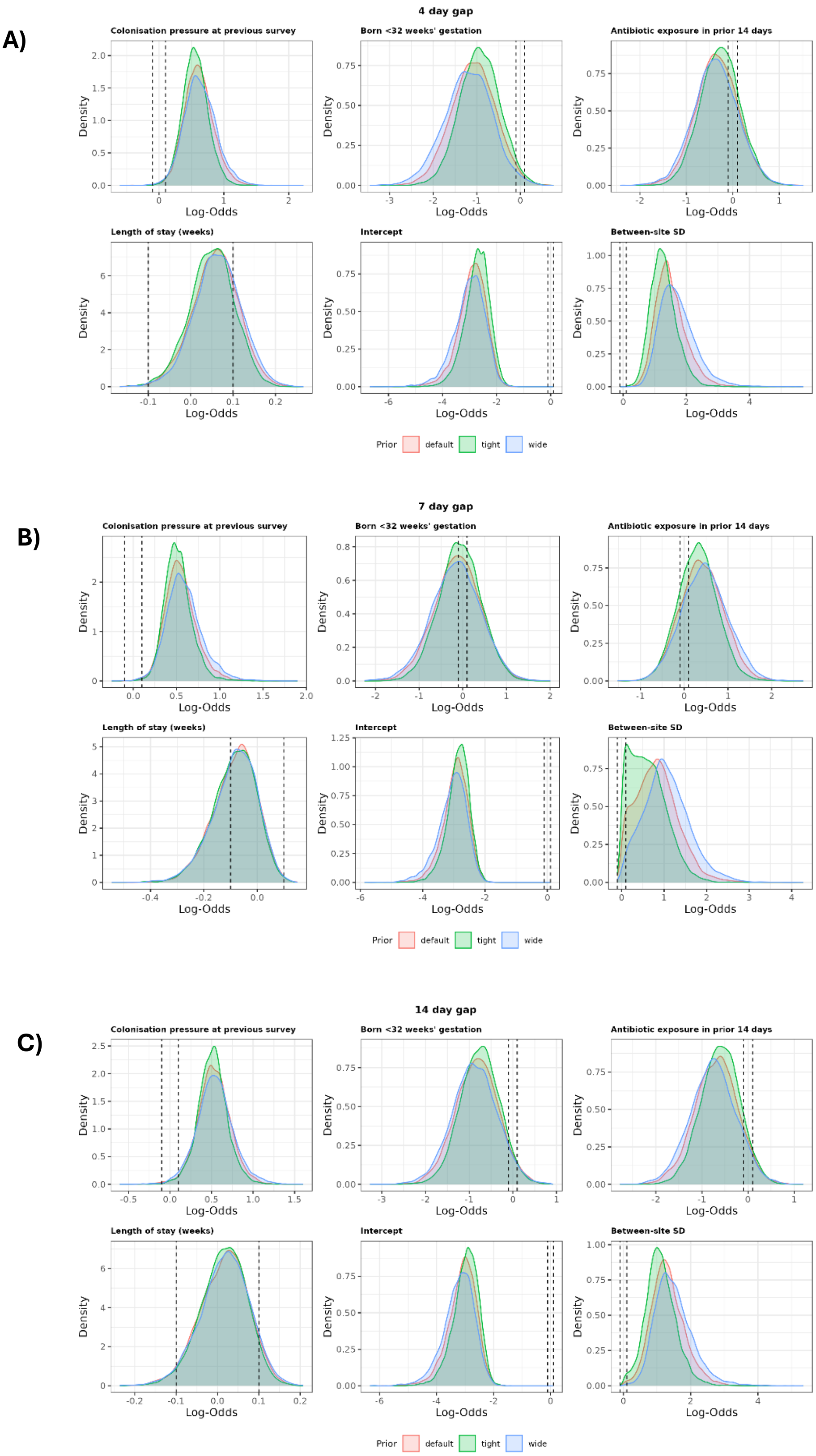

eFigure 5. Comparison of Posterior Distribution for Covariates and Between Site (Neonatal Unit) SD When Using the 3 Different Trialed Priors for the Main Analysis Population for A) model fitting dataset using colonisation pressure at survey 4 days prior, B) colonisation pressure at survey 7 days prior and C) colonisation pressure at survey 14 days prior.

© 2026 Cook A et al. *JAMA Network Open*.

Posterior checks

We conducted posterior predictive checks comparing observed acquisition outcomes to data simulated from the fitted models. The observed acquisition proportion lies within the posterior predictive distribution, indicating adequate model fit. Posterior predictive checks stratified by site demonstrate that the model adequately captures between-site variation in acquisition rates (eFigure 6).

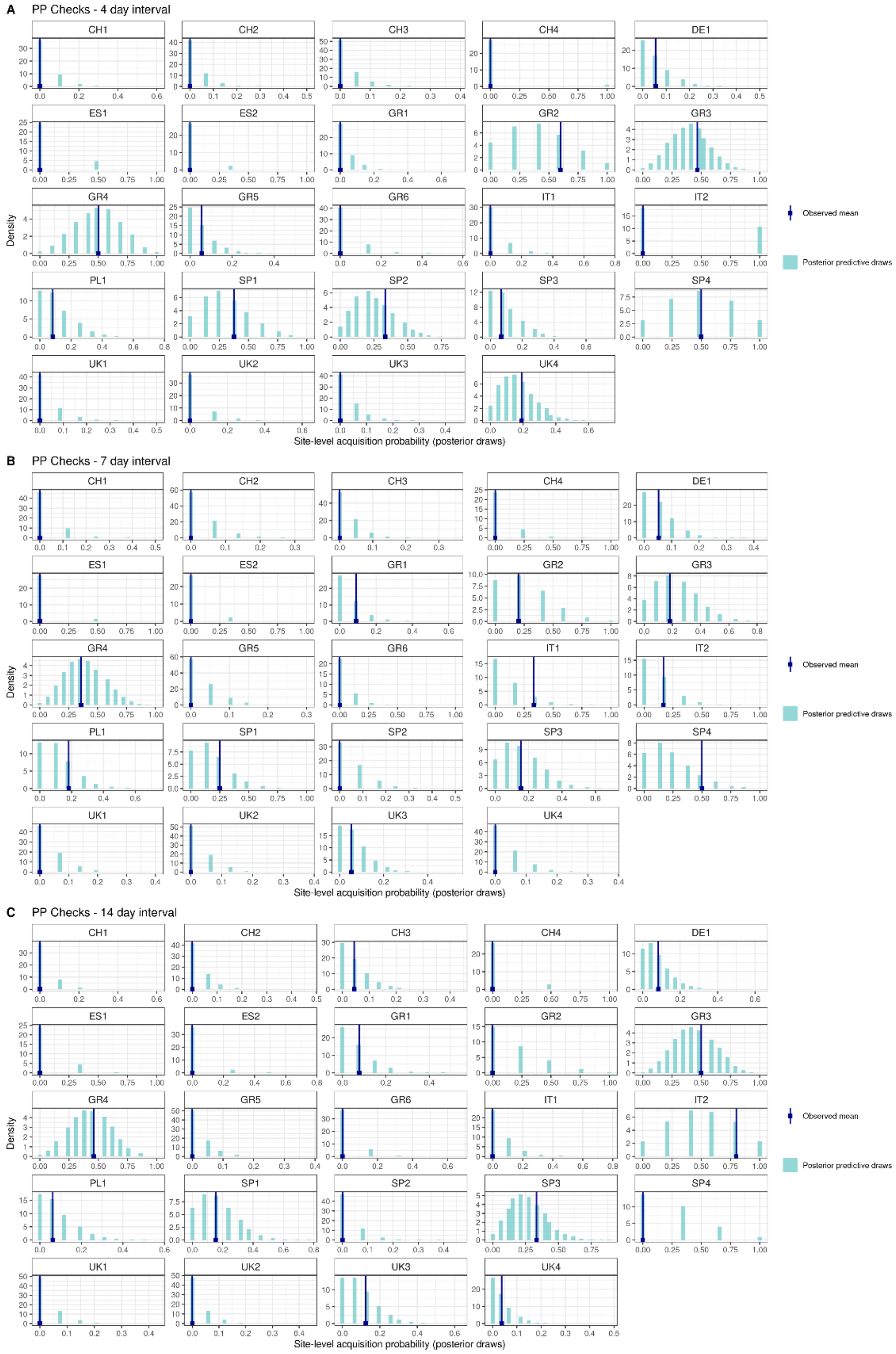

eFigure 6. Posterior Predictive Checks of the Main 3 Models (4-, 7-, and 14-Day Intervals) Showing Posterior Predictive Draws of ESBL Colonization and the Observed Mean Probability of ESBL Colonszation by Neonatal Unit

© 2026 Cook A et al. *JAMA Network Open*.

Sensitivity analysis

We restricted the model fitting datasets to only infants in neonatal units that had at least one colonised infant in all survey time points (10 sites). We re-fit the models on these restricted datasets with the intervals from previous surveys. We conducted the same prior checks for these models. Based on these checks, we used our default priors for model fitting of:

- Coefficients: *Normal*(mean = 0, sd = 1.5)
- Intercept: *Student t*(df = 3, location = -2, scale = 1.5)
- Between Unit SD: *Exponential*(rate = 1)

© 2026 Cook A et al. *JAMA Network Open*.

Results

Overall study population

eTable 5. Table of Overall Study Population Including all Infants Surveyed by Survey Number. Note: percentages are column percentages (per survey)

Characteristic	Survey Number 1 N = 466[1]	Survey Number 2 N = 453[1]	Survey Number 3 N = 464[1]	Survey Number 4 N = 464[1]
**Neonatal Unit**				
CH1	16 (3.4%)	14 (3.1%)	12 (2.6%)	11 (2.4%)
CH2	21 (4.5%)	20 (4.4%)	17 (3.7%)	17 (3.7%)
CH3	32 (6.9%)	27 (6.0%)	27 (5.8%)	30 (6.5%)
CH4	3 (0.6%)	5 (1.1%)	6 (1.3%)	5 (1.1%)
DE1	33 (7.1%)	27 (6.0%)	29 (6.3%)	36 (7.8%)
ES1	2 (0.4%)	2 (0.4%)	5 (1.1%)	4 (0.9%)
ES2	4 (0.9%)	6 (1.3%)	5 (1.1%)	5 (1.1%)
GR1	12 (2.6%)	13 (2.9%)	13 (2.8%)	14 (3.0%)
GR2	10 (2.1%)	10 (2.2%)	13 (2.8%)	9 (1.9%)
GR3	20 (4.3%)	22 (4.9%)	20 (4.3%)	18 (3.9%)
GR4	22 (4.7%)	24 (5.3%)	23 (5.0%)	19 (4.1%)
GR5	21 (4.5%)	22 (4.9%)	23 (5.0%)	22 (4.7%)
GR6	13 (2.8%)	13 (2.9%)	13 (2.8%)	12 (2.6%)
IT1	21 (4.5%)	17 (3.8%)	17 (3.7%)	19 (4.1%)
IT2	7 (1.5%)	10 (2.2%)	7 (1.5%)	7 (1.5%)
PL1	37 (7.9%)	32 (7.1%)	35 (7.5%)	37 (8.0%)
SP1	16 (3.4%)	17 (3.8%)	15 (3.2%)	19 (4.1%)
SP2	19 (4.1%)	22 (4.9%)	17 (3.7%)	17 (3.7%)
SP3	25 (5.4%)	30 (6.6%)	33 (7.1%)	33 (7.1%)
SP4	9 (1.9%)	7 (1.5%)	7 (1.5%)	10 (2.2%)
UK1	26 (5.6%)	23 (5.1%)	26 (5.6%)	26 (5.6%)
UK2	15 (3.2%)	16 (3.5%)	21 (4.5%)	23 (5.0%)
UK3	30 (6.4%)	29 (6.4%)	28 (6.0%)	23 (5.0%)
UK4	52 (11%)	45 (9.9%)	52 (11%)	48 (10%)
**Gestational age (weeks)**	33 (28 - 37)	32 (27 - 36)	32 (28 - 37)	32 (28 - 37)
**Birthweight (grams)**	1,728 (997 - 2,840)	1,650 (970 - 2,640)	1,690 (985 - 2,710)	1,700 (950 - 2,660)
**Postnatal age (days) at survey**	18 (7 - 44)	20 (8 - 42)	20 (7 - 45)	19 (8 - 43)
**Length of stay on NICU at survey (days)**	13 (4 - 31)	14 (5 - 32)	14 (5 - 34)	15 (5 - 33)
**Birth status**				
Hospitalised since birth	434 (94%)	424 (94%)	431 (93%)	437 (95%)
Not hospitalised since birth	30 (6.5%)	29 (6.4%)	29 (6.3%)	24 (5.2%)
Unknown	0 (0%)	0 (0%)	1 (0.2%)	0 (0%)
**Surgery prior to survey**				
No	356 (76%)	344 (76%)	359 (77%)	365 (79%)
Yes	103 (22%)	102 (23%)	103 (22%)	95 (20%)
Unknown	7 (1.5%)	7 (1.5%)	2 (0.4%)	4 (0.9%)
**Kangaroo Care in prior 24 hours to survey**				
No	210 (45%)	205 (45%)	183 (39%)	169 (36%)
Yes	224 (48%)	229 (51%)	263 (57%)	261 (56%)
Unknown	32 (6.9%)	19 (4.2%)	18 (3.9%)	34 (7.3%)
**Enteral breast milk in prior 24 hours**				
No	135 (29%)	129 (28%)	121 (26%)	120 (26%)
Yes	269 (58%)	265 (58%)	287 (62%)	291 (63%)
Unknown	62 (13%)	59 (13%)	56 (12%)	53 (11%)
**Presence of central line in prior 24 hours**				
No	361 (77%)	341 (75%)	344 (74%)	370 (80%)
Yes	104 (22%)	112 (25%)	119 (26%)	93 (20%)
Unknown	1 (0.2%)	0 (0%)	1 (0.2%)	1 (0.2%)
**Receipt of TPN in prior 24 hours**				

© 2026 Cook A et al. *JAMA Network Open*.

Characteristic	Survey Number 1<br>N = 466[1]	2<br>N = 453[1]	3<br>N = 464[1]	4<br>N = 464[1]
No	351 (75%)	344 (76%)	347 (76%)	372 (81%)
Yes	112 (24%)	108 (24%)	111 (24%)	89 (19%)
Unknown	2 (0.4%)	0 (0%)	0 (0%)	1 (0.2%)
**Invasive ventilation in prior 24 hours**				
No	415 (89%)	402 (89%)	417 (90%)	422 (91%)
Yes	48 (10%)	51 (11%)	45 (9.7%)	40 (8.6%)
Unknown	3 (0.6%)	0 (0%)	2 (0.4%)	2 (0.4%)
**Antibiotic exposure within 14 days prior to survey**				
No	218 (47%)	205 (45%)	195 (42%)	191 (41%)
Yes	246 (53%)	247 (55%)	268 (58%)	271 (58%)
Unknown	2 (0.4%)	1 (0.2%)	1 (0.2%)	2 (0.4%)
**Number of infants with any resistant bacterial gene of interest isolated from stool sample**				
Not colonised	293 (63%)	283 (62%)	292 (63%)	272 (59%)
Colonised	67 (14%)	74 (16%)	78 (17%)	89 (19%)
Unknown	106 (23%)	96 (21%)	94 (20%)	103 (22%)
**Number of infants colonised with any resistant bacterial gene of interest (stool or skin sample)**				
Not colonised	286 (61%)	276 (61%)	280 (60%)	267 (58%)
Colonised	76 (16%)	81 (18%)	88 (19%)	94 (20%)
Unknown	104 (22%)	96 (21%)	96 (21%)	103 (22%)
**Number of infants colonised with ESBL genes**				
Not colonised	315 (68%)	305 (67%)	317 (68%)	306 (66%)
Colonised	45 (9.7%)	52 (11%)	53 (11%)	55 (12%)
Unknown	106 (23%)	96 (21%)	94 (20%)	103 (22%)
**Number of infants colonised with carbapenemase genes**				
Not colonised	339 (73%)	333 (74%)	343 (74%)	332 (72%)
Colonised	21 (4.5%)	24 (5.3%)	27 (5.8%)	29 (6.3%)
Unknown	106 (23%)	96 (21%)	94 (20%)	103 (22%)
**Number of infants colonised with vancomycin resistance genes**				
Not colonised	349 (75%)	345 (76%)	350 (75%)	330 (71%)
Colonised	11 (2.4%)	12 (2.6%)	20 (4.3%)	31 (6.7%)
Unknown	106 (23%)	96 (21%)	94 (20%)	103 (22%)
**Number of infants colonised with MRSA genes**				
Not colonised	451 (97%)	441 (97%)	447 (96%)	455 (98%)
Colonised	10 (2.1%)	8 (1.8%)	12 (2.6%)	6 (1.3%)
Unknown	5 (1.1%)	4 (0.9%)	5 (1.1%)	3 (0.6%)
**Number of infants colonised with MRCoNS genes**				
Not colonised	31 (6.7%)	27 (6.0%)	46 (9.9%)	32 (6.9%)
Colonised	430 (92%)	422 (93%)	413 (89%)	429 (92%)
Unknown	5 (1.1%)	4 (0.9%)	5 (1.1%)	3 (0.6%)

[1]n (%); Median (Q1 - Q3); Percentages are column percentages where each PPS sums to 100%; NICU = neonatal intensive care unit; **TPN:** total parenteral nutrition; ESBL = extended-spectrum beta-lactamase; MRSA = methicillin-resistant *Staphylococcus aureus*; MRCoNS = methicillin-resistant Coagulase-negative Staphylococci

© 2026 Cook A et al. *JAMA Network Open*.

Microbiology findings

eTable 6. Stool Resistant Bacterial Colonization Patterns Detected in the Study.

**Stool sample results**	
**Total infant-survey observations**	1847
**Infant-surveys with available stool colonisation results (n, %)**	1448 (78)*
**Infant-surveys with any positive stool colonisation (n, % of total available samples)**	308 (21%)
**Antibiotic resistance patterns**	n (% of positive samples)
ESBL genes detected	205 (67%)
Carbapenemase genes detected	101 (33%)
Vancomycin resistance genes detected	74 (24%)
Combinations	
ESBL & carbapenemase genes	33 (11%)
ESBL & vancomycin resistance genes	13 (4%)
Carbapenemase & vancomycin resistance genes	10 (3%)
ESBL & carbapenemase & vancomycin resistance genes	8 (3%)

**Infant-surveys without available stool colonisation results are predominantly due to unavailability of stool samples (i.e. infant did not produce stool sample). ESBL = extended-spectrum beta-lactamase*

© 2026 Cook A et al. *JAMA Network Open*.

eTable 7. Antibiotic Resistance Genes Detected in Available Stool Collected Samples by Survey Number. Note samples may not have been collected if infant did not produce a stool or was unavailable on the survey day.

			Survey Number			
**PCR Type**	**Total Samples**	**PCR Result**	**1**	**2**	**3**	**4**
**Total stool samples by PPS**	**1,448**	**-**	**360**	**357**	**370**	**361**
blaCTX-M Group 1		Negative	315 (87.5%)	306 (85.7%)	317 (85.7%)	307 (85%)
		Positive	45 (12.5%)	51 (14.3%)	53 (14.3%)	54 (15%)
blaCTX-M Group 9		Negative	360 (100%)	356 (99.7%)	370 (100%)	360 (99.7%)
		Positive	-	1 (0.3%)	-	1 (0.3%)
SHV		Negative	239 (66.4%)	227 (63.6%)	239 (64.6%)	230 (63.7%)
		Positive	121 (33.6%)	130 (36.4%)	131 (35.4%)	131 (36.3%)
blaIMP		Negative	360 (100%)	357 (100%)	370 (100%)	361 (100%)
blaKPC		Negative	349 (96.9%)	345 (96.6%)	353 (95.4%)	347 (96.1%)
		Positive	11 (3.1%)	12 (3.4%)	17 (4.6%)	14 (3.9%)
blaNDM		Negative	357 (99.2%)	357 (100%)	370 (100%)	360 (99.7%)
		Positive	3 (0.8%)	-	-	1 (0.3%)
blaVIM		Negative	349 (96.9%)	344 (96.4%)	350 (94.6%)	346 (95.8%)
		Positive	11 (3.1%)	13 (3.6%)	20 (5.4%)	15 (4.2%)
blaOXA-48		Negative	357 (99.2%)	355 (99.4%)	369 (99.7%)	357 (98.9%)
		Positive	3 (0.8%)	2 (0.6%)	1 (0.3%)	4 (1.1%)
VanB		Negative	360 (100%)	357 (100%)	370 (100%)	360 (99.7%)
		Positive	-	-	-	1 (0.3%)
VanA		Negative	349 (96.9%)	345 (96.6%)	350 (94.6%)	331 (91.7%)
		Positive	11 (3.1%)	12 (3.4%)	20 (5.4%)	30 (8.3%)
**Total skin swabs by PPS**	**1,830**	**-**	**461**	**449**	**459**	**461**
mecA/mecC		Negative	21 (4.6%)	19 (4.2%)	34 (7.4%)	26 (5.6%)
		Positive	440 (95.4%)	430 (95.8%)	425 (92.6%)	435 (94.4%)
SCCmec/orfX		Negative	451 (97.8%)	441 (98.2%)	446 (97.2%)	455 (98.7%)
		Positive	10 (2.2%)	8 (1.8%)	13 (2.8%)	6 (1.3%)

© 2026 Cook A et al. *JAMA Network Open*.

eTable 8. Antibiotic Resistance Gene Targets Detected in Collected Stool Samples and Skin Swabs by Neonatal Unit.

PCR Type	Total Samples	PCR Result	CH1	CH2	CH3	CH4	DE1	ES1	ES2	GR1	GR2	GR3	GR4	GR5	GR6	IT1	IT2	PL1	SP1	SP2	SP3	SP4	UK1	UK2	UK3	UK4
Total stool samples by site	1,448		44	72	99	10	99	13	20	49	27	69	82	83	34	43	27	101	54	66	96	31	63	56	90	120
**blaCTX-M Group 1**		**Neg**	43 (97.7%)	72 (100%)	98 (99%)	10 (100%)	93 (93.9%)	13 (100%)	20 (100%)	46 (93.9%)	19 (70.4%)	46 (66.7%)	38 (46.3%)	81 (97.6%)	34 (100%)	41 (95.3%)	15 (55.6%)	86 (85.1%)	40 (74.1%)	59 (89.4%)	71 (74%)	15 (48.4%)	63 (100%)	56 (100%)	80 (88.9%)	106 (88.3%)
		**Pos**	1 (2.3%)	-	1 (1%)	-	6 (6.1%)	-	-	3 (6.1%)	8 (29.6%)	23 (33.3%)	44 (53.7%)	2 (2.4%)	-	2 (4.7%)	12 (44.4%)	15 (14.9%)	14 (25.9%)	7 (10.6%)	25 (26%)	16 (51.6%)	-	-	10 (11.1%)	14 (11.7%)
**blaCTX-M Group 9**		**Neg**	44 (100%)	72 (100%)	99 (100%)	10 (100%)	99 (100%)	13 (100%)	20 (100%)	49 (100%)	27 (100%)	69 (100%)	82 (100%)	83 (100%)	34 (100%)	43 (100%)	27 (100%)	100 (99%)	54 (100%)	66 (100%)	95 (99%)	31 (100%)	63 (100%)	56 (100%)	90 (100%)	120 (100%)
		**Pos**	-	-	-	-	-	-	-	-	-	-	-	-	-	-	-	1 (1%)	-	-	1 (1%)	-	-	-	-	-
**SHV**		**Neg**	41 (93.2%)	52 (72.2%)	67 (67.7%)	10 (100%)	63 (63.6%)	9 (69.2%)	14 (70%)	34 (69.4%)	16 (59.3%)	10 (14.5%)	25 (30.5%)	62 (74.7%)	25 (73.5%)	40 (93%)	16 (59.3%)	55 (54.5%)	16 (29.6%)	50 (75.8%)	50 (52.1%)	19 (61.3%)	50 (79.4%)	47 (83.9%)	65 (72.2%)	99 (82.5%)
		**Pos**	3 (6.8%)	20 (27.8%)	32 (32.3%)	-	36 (36.4%)	4 (30.8%)	6 (30%)	15 (30.6%)	11 (40.7%)	59 (85.5%)	57 (69.5%)	21 (25.3%)	9 (26.5%)	3 (7%)	11 (40.7%)	46 (45.5%)	38 (70.4%)	16 (24.2%)	46 (47.9%)	12 (38.7%)	13 (20.6%)	9 (16.1%)	25 (27.8%)	21 (17.5%)
**blaIMP**		**Neg**	44 (100%)	72 (100%)	99 (100%)	10 (100%)	99 (100%)	13 (100%)	20 (100%)	49 (100%)	27 (100%)	69 (100%)	82 (100%)	83 (100%)	34 (100%)	43 (100%)	27 (100%)	101 (100%)	54 (100%)	66 (100%)	96 (100%)	31 (100%)	63 (100%)	56 (100%)	90 (100%)	120 (100%)
**blaKPC**		**Neg**	44 (100%)	72 (100%)	99 (100%)	10 (100%)	99 (100%)	13 (100%)	20 (100%)	49 (100%)	26 (96.3%)	22 (31.9%)	82 (100%)	79 (95.2%)	34 (100%)	43 (100%)	27 (100%)	101 (100%)	54 (100%)	65 (98.5%)	95 (99%)	31 (100%)	63 (100%)	56 (100%)	90 (100%)	120 (100%)
		**Pos**	-	-	-	-	-	-	-	-	1 (3.7%)	47 (68.1%)	-	4 (4.8%)	-	-	-	-	-	1 (1.5%)	1 (1%)	-	-	-	-	-

© 2026 Cook A et al. *JAMA Network Open*.

PCR Type	Total Samples	PCR Result	CH1	CH2	CH3	CH4	DE1	ES1	ES2	GR1	GR2	GR3	GR4	GR5	GR6	IT1	IT2	PL1	SP1	SP2	SP3	SP4	UK1	UK2	UK3	UK4
**blaNDM**		**Neg**	44 (100%)	72 (100%)	99 (100%)	10 (100%)	99 (100%)	13 (100%)	20 (100%)	47 (95.9%)	27 (100%)	69 (100%)	82 (100%)	83 (100%)	34 (100%)	41 (95.3%)	27 (100%)	101 (100%)	54 (100%)	66 (100%)	96 (100%)	31 (100%)	63 (100%)	56 (100%)	90 (100%)	120 (100%)
		**Pos**	-	-	-	-	-	-	-	2 (4.1%)	-	-	-	-	-	2 (4.7%)	-	-	-	-	-	-	-	-	-	-
**blaVIM**		**Neg**	43 (97.7%)	72 (100%)	99 (100%)	10 (100%)	99 (100%)	13 (100%)	20 (100%)	49 (100%)	22 (81.5%)	41 (59.4%)	76 (92.7%)	82 (98.8%)	34 (100%)	41 (95.3%)	27 (100%)	99 (98%)	54 (100%)	65 (98.5%)	96 (100%)	18 (58.1%)	63 (100%)	56 (100%)	90 (100%)	120 (100%)
		**Pos**	1 (2.3%)	-	-	-	-	-	-	-	5 (18.5%)	28 (40.6%)	6 (7.3%)	1 (1.2%)	-	2 (4.7%)	-	2 (2%)	-	1 (1.5%)	-	13 (41.9%)	-	-	-	-
**blaOXA-48**		**Neg**	44 (100%)	72 (100%)	98 (99%)	10 (100%)	99 (100%)	13 (100%)	20 (100%)	49 (100%)	27 (100%)	68 (98.6%)	82 (100%)	83 (100%)	34 (100%)	41 (95.3%)	27 (100%)	99 (98%)	53 (98.1%)	66 (100%)	95 (99%)	31 (100%)	63 (100%)	55 (98.2%)	90 (100%)	119 (99.2%)
		**Pos**	-	-	1 (1%)	-	-	-	-	-	-	1 (1.4%)	-	-	-	2 (4.7%)	-	2 (2%)	1 (1.9%)	-	1 (1%)	-	-	1 (1.8%)	-	1 (0.8%)
**VanB**		**Neg**	44 (100%)	72 (100%)	99 (100%)	10 (100%)	99 (100%)	13 (100%)	20 (100%)	49 (100%)	27 (100%)	69 (100%)	82 (100%)	83 (100%)	34 (100%)	43 (100%)	27 (100%)	100 (99%)	54 (100%)	66 (100%)	96 (100%)	31 (100%)	63 (100%)	56 (100%)	90 (100%)	120 (100%)
		**Pos**	-	-	-	-	-	-	-	-	-	-	-	-	-	-	-	1 (1%)	-	-	-	-	-	-	-	-
**VanA**		**Neg**	44 (100%)	72 (100%)	99 (100%)	10 (100%)	98 (99%)	13 (100%)	20 (100%)	32 (65.3%)	20 (74.1%)	56 (81.2%)	65 (79.3%)	79 (95.2%)	24 (70.6%)	43 (100%)	27 (100%)	99 (98%)	52 (96.3%)	66 (100%)	96 (100%)	31 (100%)	63 (100%)	56 (100%)	90 (100%)	120 (100%)
		**Pos**	-	-	-	-	1 (1%)	-	-	17 (34.7%)	7 (25.9%)	13 (18.8%)	17 (20.7%)	4 (4.8%)	10 (29.4%)	-	-	2 (2%)	2 (3.7%)	-	-	-	-	-	-	-
**Total skin swabs by site**	**1,830**		**53**	**75**	**113**	**19**	**124**	**13**	**20**	**52**	**42**	**80**	**87**	**88**	**51**	**74**	**31**	**140**	**67**	**75**	**120**	**33**	**93**	**75**	**110**	**195**

© 2026 Cook A et al. *JAMA Network Open*.

PCR Type	Total Samples	PCR Result	CH1	CH2	CH3	CH4	DE1	ES1	ES2	GR1	GR2	GR3	GR4	GR5	GR6	IT1	IT2	PL1	SP1	SP2	SP3	SP4	UK1	UK2	UK3	UK4
**mecA/ mecC**		**Neg**	1 (1.9%)	7 (9.3%)	14 (12.4%)	7 (36.8%)	15 (12.1%)	-	3 (15%)	-	1 (2.4%)	-	2 (2.3%)	1 (1.1%)	6 (11.8%)	7 (9.5%)	1 (3.2%)	5 (3.6%)	3 (4.5%)	3 (4%)	5 (4.2%)	-	5 (5.4%)	1 (1.3%)	2 (1.8%)	11 (5.6%)
		**Pos**	52 (98.1%)	68 (90.7%)	99 (87.6%)	12 (63.2%)	109 (87.9%)	13 (100%)	17 (85%)	52 (100%)	41 (97.6%)	80 (100%)	85 (97.7%)	87 (98.9%)	45 (88.2%)	67 (90.5%)	30 (96.8%)	135 (96.4%)	64 (95.5%)	72 (96%)	115 (95.8%)	33 (100%)	88 (94.6%)	74 (98.7%)	108 (98.2%)	184 (94.4%)
**SCCmec/ orfX**		**Neg**	43 (81.1%)	75 (100%)	111 (98.2%)	19 (100%)	120 (96.8%)	13 (100%)	20 (100%)	50 (96.2%)	37 (88.1%)	78 (97.5%)	86 (98.9%)	87 (98.9%)	51 (100%)	74 (100%)	30 (96.8%)	139 (99.3%)	67 (100%)	74 (98.7%)	120 (100%)	33 (100%)	93 (100%)	71 (94.7%)	110 (100%)	192 (98.5%)
		**Pos**	10 (18.9%)	-	2 (1.8%)	-	4 (3.2%)	-	-	2 (3.8%)	5 (11.9%)	2 (2.5%)	1 (1.1%)	1 (1.1%)	-	-	1 (3.2%)	1 (0.7%)	-	1 (1.3%)	-	-	-	4 (5.3%)	-	3 (1.5%)

© 2026 Cook A et al. *JAMA Network Open*.

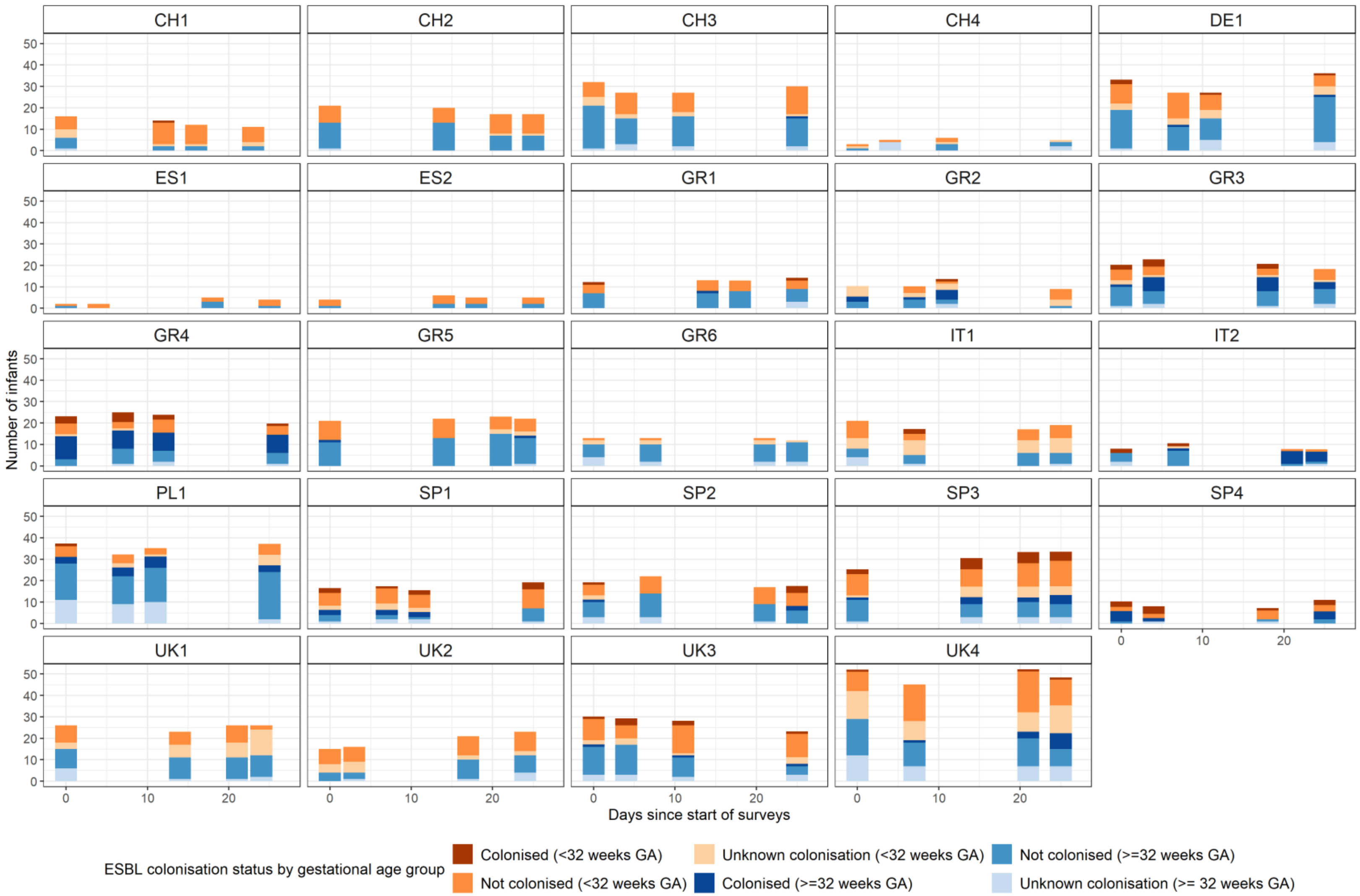

eFigure 7. Number of Infants by ESBL Colonization Status and Prematurity Status (<32 weeks, orange; or ≥32 weeks, blue).

© 2026 Cook A et al. *JAMA Network Open*.

Main analysis

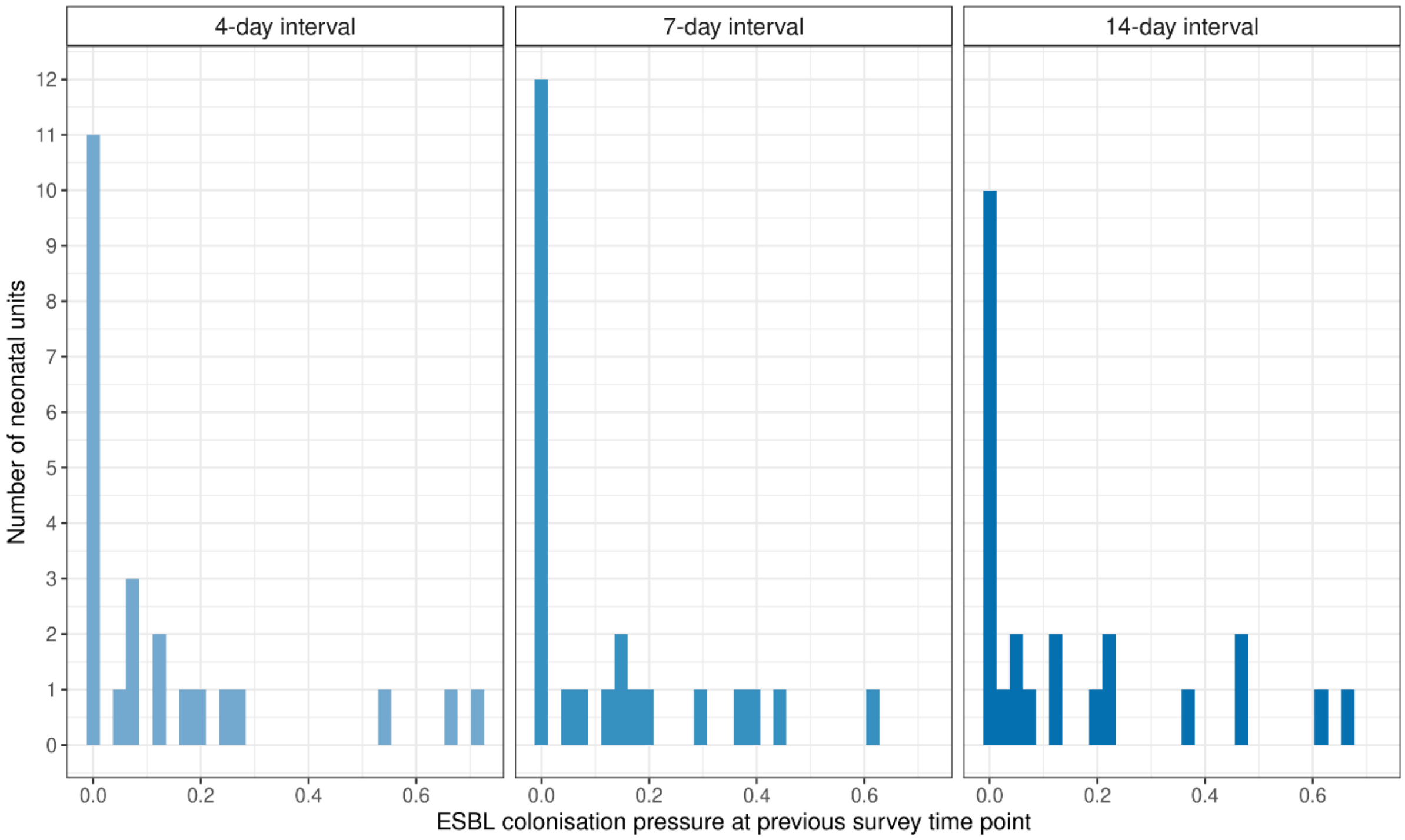

eFigure 8. Colonization Pressure (Proportion of Infants That Were ESBL-Colonized at the Previous Survey Time Point) for Each Model Fitting Dataset Where the Previous Survey Time Point Occurred 4 Days, 7 Days or 14 Days Before the Analyzed Survey.

© 2026 Cook A et al. *JAMA Network Open*.

eTable 9. Table Showing Eligibility Status for Incident Acquisition – Either Not Colonized at Previous Survey or Newly Admitted Since Previous Survey – and Colonization Status by Analysis Dataset.

Characteristic	Model 1 - 4 day interval			Model 2 - 7 day interval			Model 3 - 14 day interval		
	**Total** N = 256	**Not ESBL colonised** N = 223[1]	**ESBL colonised** N = 33[1]	**Total** N = 269	**Not ESBL colonised** N = 245[1]	**ESBL colonized** N = 24[1]	**Total** N = 304	**Not ESBL colonised** N = 271[1]	**ESBL colonised** N = 33[1]
**Eligibility status for incident acquisition**									
Present at previous survey but not colonised	202 (79%)	175 (78%)	27 (82%)	164 (61%)	151 (62%)	13 (54%)	137 (45%)	125 (46%)	12 (36%)
Admitted since previous survey	54 (21%)	48 (22%)	6 (18%)	105 (39%)	94 (38%)	11 (46%)	167 (55%)	146 (54%)	21 (66%)
**Difference in colonization by eligibility status (p (chi-square test))**		0.8331			0.6197			0.3795	
**Length of stay by eligibility status (weeks)**									
Present at previous survey but not colonised	3.4 (1.6-6.1)	3.4 (1.9-6.1)	3.4 (1.3-6.1)	3.6 (2.1 - 6.6)	3.7 (2.1-6.6)	3.4 (2-5.4)	4.4 (3-7)	4.4 (3-7.3)	5.1 (4.1-6.4)
Admitted since previous survey	0.36 (0.18 – 0.43)	0.28 (0.14 – 0.43)	0.5 (0.32-0.57)	0.43 (0.14-0.71)	0.43 (0.14-0.71)	0.43(0.29-0.57)	0.71 (0.43 – 1.29)	0.71 (0.43-1.29)	0.71 (0.57-1.29)

© 2026 Cook A et al. *JAMA Network Open*.

eTable 10. Model Coefficients (Mean Odds of Posterior Draws), 95% Credible Interval and Probability of Direction of Association for the 3 Main Analysis Models Fit Using Colonization Pressure at the Previous Survey With Intervals Between Surveys of 4 Days, 7 Days, and 14 Days.

Parameter	4-day model		7-day model		14-day model	
	Mean OR [95% CrI]	Probability of direction[1]	Mean OR [95% CrI]	Probability of direction	Mean OR [95% CrI]	Probability of direction
Intercept	0.06 [0.01, 0.16]	-	0.04 [0.01, 0.10]	-	0.06 [0.01, 0.15]	-
ESBL colonisation pressure at previous survey	1.89 [1.22, 2.94]	1.00	1.76 [1.26, 2.63]	1.00	1.73 [1.15, 2.52]	0.99
Length of stay (weeks)	1.06 [0.95, 1.18]	0.86	0.92 [0.77, 1.06]	0.85	1.02 [0.90, 1.13]	0.63
Antibiotic exposure in prior 14 days	0.79 [0.29, 1.74]	0.78	1.69 [0.58, 3.95]	0.79	0.56 [0.19, 1.22]	0.93
Born <32 weeks' gestation	0.39 [0.13, 0.90]	0.99	1.02 [0.30, 2.47]	0.58	0.49 [0.17, 1.13]	0.96
SD of Unit-Intercept (log-odds)	1.51 [0.74, 2.60]	-	0.83 [0.05, 1.89]	-	1.32 [0.45, 2.43]	-

[1]Probability of direction is the certainty that the effect goes in the direction shown in the coefficient (i.e. positive or negative)

© 2026 Cook A et al. *JAMA Network Open*.

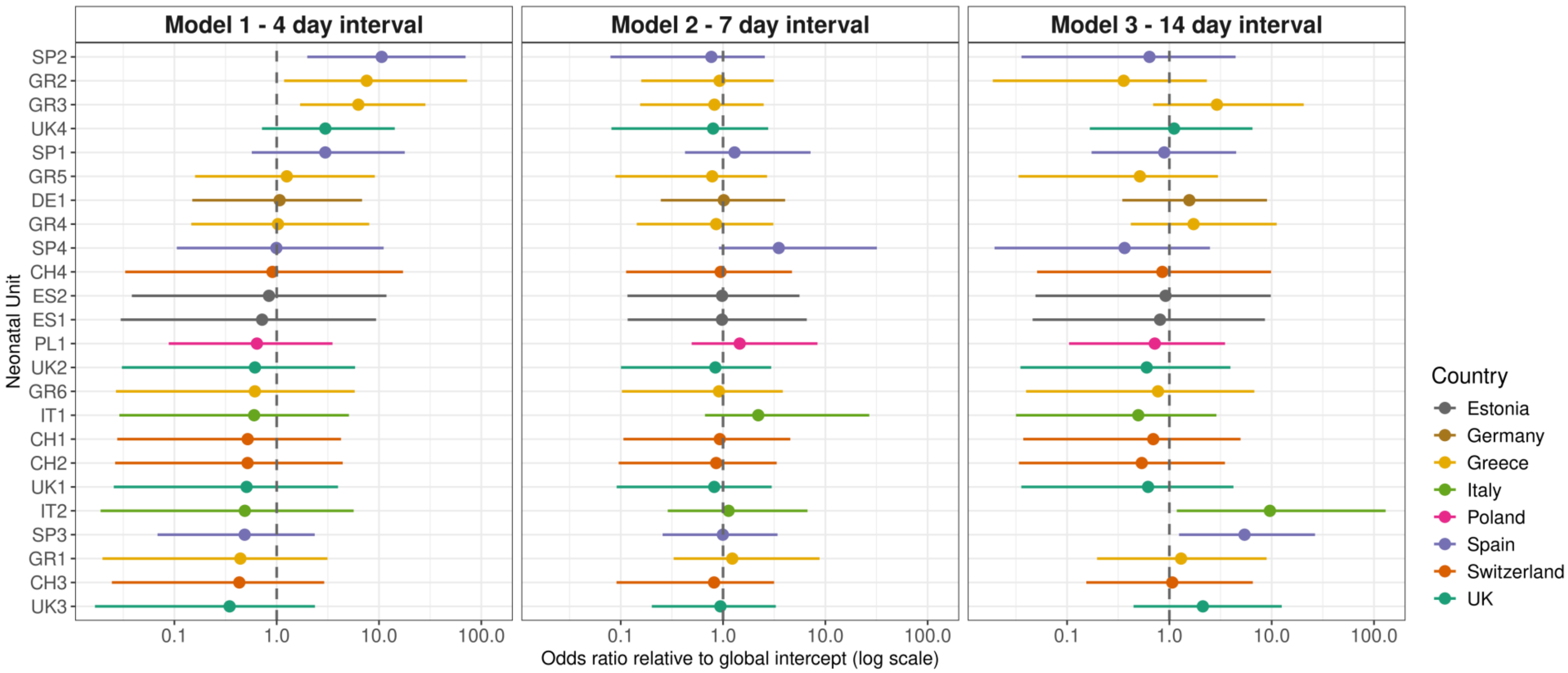

eFigure 9. Neonatal Unit Random Effects Odds Ratio Deviations From the Global Intercept (Mean and 95% Credible Interval) of 3 Models. Note the x-axis is on the log scale for plotting. Models were fit on three datasets where colonisation pressure was measured from the previous survey (4 days, 7 days and 14 days before respectively).

© 2026 Cook A et al. *JAMA Network Open*.

Sensitivity analysis

eTable 11. Characteristics of Infants Included in the Sensitivity Analysis Restricted to Neonatal Units With Colonization Pressure >0 at all 4 Surveys for Each Model-Fitting Dataset for 4-Day Interval, 7-Day Interval, and 14-Day Interval.

Characteristic	Model 1 - 4 day interval		Model 2 - 7 day interval		Model 3 - 14 day interval	
	**Not ESBL colonised** N = 98[1]	**ESBL colonised** N = 24[1]	**Not ESBL colonised** N = 105[1]	**ESBL colonised** N = 20[1]	**Not ESBL colonised** N = 118[1]	**ESBL colonised** N = 31[1]
**Country**						
Germany	17 (17.3%)	1 (4.2%)	18 (17.1%)	1 (5.0%)	22 (18.6%)	2 (6.5%)
Greece	13 (13.3%)	12 (50.0%)	18 (17.1%)	7 (35.0%)	14 (11.9%)	13 (41.9%)
Italy	1 (1.0%)	0 (0%)	5 (4.8%)	1 (5.0%)	1 (0.8%)	4 (12.9%)
Poland	11 (11.2%)	1 (4.2%)	9 (8.6%)	2 (10.0%)	15 (12.7%)	1 (3.2%)
Spain	21 (21.4%)	6 (25.0%)	21 (20.0%)	8 (40.0%)	26 (22.0%)	8 (25.8%)
UK	35 (35.7%)	4 (16.7%)	34 (32.4%)	1 (5.0%)	40 (33.9%)	3 (9.7%)
**Gestational age (weeks)**	33.0 (28.0 - 37.0)	32.0 (30.5 - 37.0)	33.0 (29.0 - 38.0)	37.0 (31.5 - 38.5)	33.0 (28.0 - 37.0)	33.0 (31.0 - 36.0)
**Gestational age group**						
<28 weeks	21 (21.4%)	3 (12.5%)	20 (19.0%)	1 (5.0%)	25 (21.2%)	3 (9.7%)
28 - <32 weeks	23 (23.5%)	4 (16.7%)	26 (24.8%)	4 (20.0%)	29 (24.6%)	6 (19.4%)
32 - <37 weeks	24 (24.5%)	10 (41.7%)	27 (25.7%)	4 (20.0%)	33 (28.0%)	15 (48.4%)
≥37 weeks	30 (30.6%)	7 (29.2%)	32 (30.5%)	11 (55.0%)	31 (26.3%)	7 (22.6%)
**Infant born <32 weeks gestation**						
<32 weeks	44 (44.9%)	7 (29.2%)	46 (43.8%)	5 (25.0%)	54 (45.8%)	9 (29.0%)
≥32 weeks	54 (55.1%)	17 (70.8%)	59 (56.2%)	15 (75.0%)	64 (54.2%)	22 (71.0%)
**Birthweight (grams)**	1,650 (930 - 2,950)	1,865 (1,365 - 2,540)	1,830 (1,100 - 2,910)	2,345 (1,715 - 3,265)	1,813 (1,040 - 2,900)	1,750 (1,440 - 2,540)
**Postnatal age (days)**	26 (7 - 52)	17 (8 - 54)	20 (6 - 49)	8 (4 - 39)	16 (6 - 45)	13 (7 - 41)
**Length of stay on NICU at survey (weeks)**	3.1 (0.7 - 5.1)	1.9 (0.7 - 6.1)	2.1 (0.4 - 4.6)	0.8 (0.4 - 3.6)	1.6 (0.6 - 4.7)	1.7 (0.7 - 4.4)
**Antibiotic exposure within 14 days**	57 (58.2%)	12 (50.0%)	59 (56.2%)	9 (45.0%)	72 (61.0%)	14 (45.2%)
**ESBL colonisation pressure at previous survey**	0.11 (0.08 - 0.25)	0.18 (0.18 - 0.55)	0.15 (0.07 - 0.36)	0.31 (0.17 - 0.53)	0.12 (0.06 - 0.21)	0.22 (0.13 - 0.47)

[1]n (%); Median (Q1 - Q3)

© 2026 Cook A et al. *JAMA Network Open*.

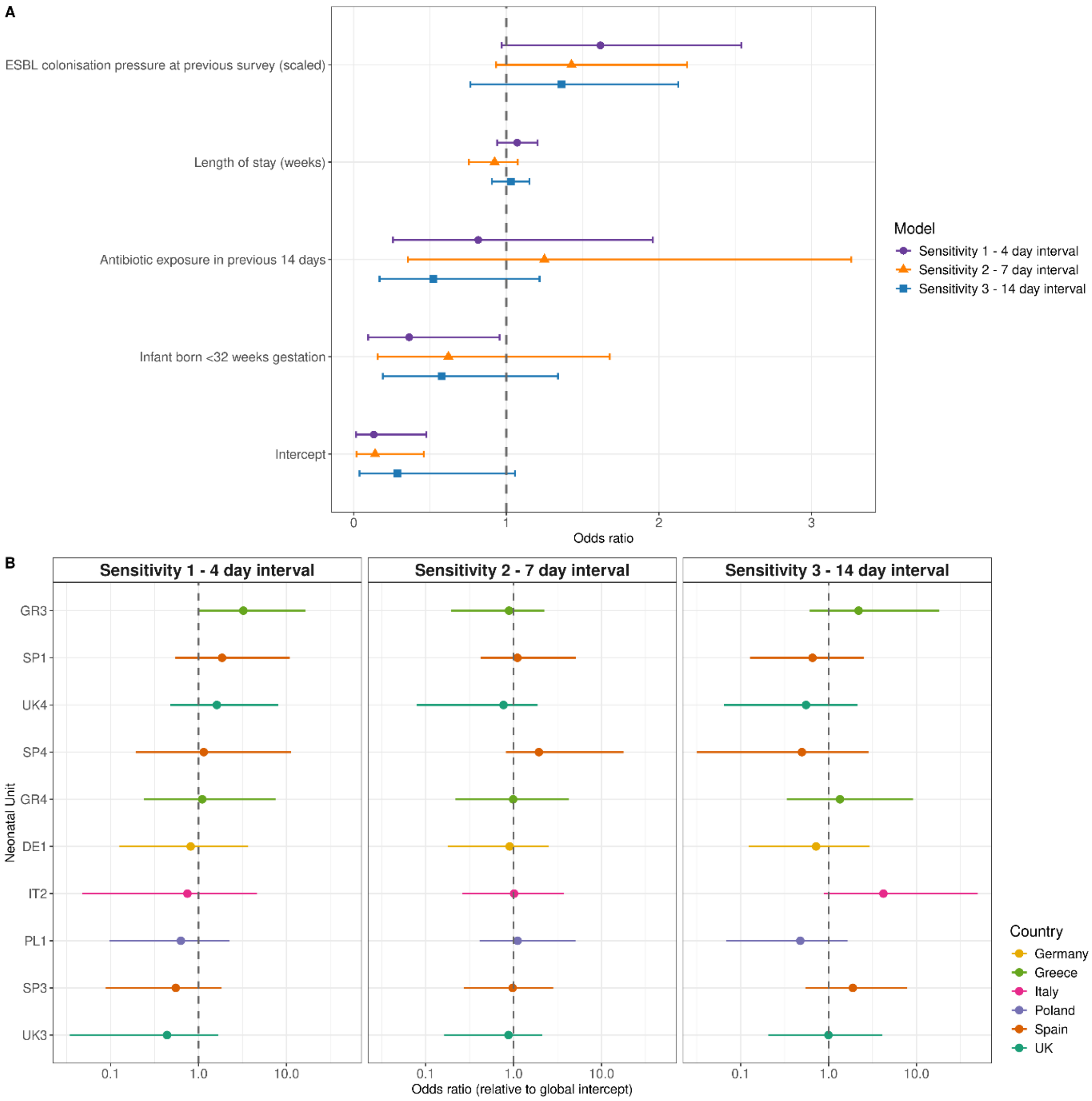

eFigure 10. A) Odds Ratio Estimates (Mean and 95% Credible Interval) of Model Fixed Effects of 3 Sensitivity Analysis Models. Note: ESBL colonisation pressure at the previous survey was multiplied by 10 prior to the analysis so that coefficients represent the effect per 0.1 increase on the original proportional scale. B) Neonatal unit random effects odds ratio deviations from the global intercept (mean and 95% credible interval) of three models. Note the x-axis is on the log scale for plotting. Models were fit on three datasets where colonisation pressure was measured from the previous survey (4 days, 7 days and 14 days before respectively).

© 2026 Cook A et al. *JAMA Network Open*.

eTable 12. Model Coefficients (Mean Odds of Posterior Draws), 95% Credible Interval and Probability of Direction of Association for the 3 Sensitivity Analysis Models Fit Using Colonization Pressure at the Previous Survey With Intervals Between Surveys of 4 Days, 7 Days, and 14 Days. Sensitivity analysis datasets were restricted to only neonatal units with at least one infant ESBL colonised at each timepoint.

Parameter	4-day model		7-day model		14-day model	
	**Mean [95% CrI]**	**Probability of direction[1]**	**Mean [95% CrI]**	**Probability of direction**	**Mean [95% CrI]**	**Probability of direction**
Intercept	0.13 [0.02, 0.48]	-	0.14 [0.02, 0.46]	-	0.29 [0.04, 1.06]	-
ESBL colonisation pressure at previous survey	1.62 [0.97, 2.54]	0.97	1.43 [0.93, 2.18]	0.96	1.36 [0.76, 2.13]	0.86
Length of stay (weeks)	1.07 [0.94, 1.20]	0.86	0.92 [0.75, 1.07]	0.83	1.03 [0.91, 1.15]	0.69
Antibiotic exposure in prior 14 days	0.82 [0.26, 1.96]	0.74	1.25 [0.35, 3.26]	0.54	0.52 [0.17, 1.22]	0.94
Born <32 weeks' gestation	0.36 [0.09, 0.96]	0.98	0.62 [0.16, 1.68]	0.87	0.58 [0.19, 1.34]	0.92
SD of Unit-Intercept (log-odds)	1.05 [0.17, 2.26]	-	0.66 [0.03, 1.76]	-	1.13 [0.20, 2.37]	-

[1]Probability of direction is the certainty that the effect goes in the direction shown in the coefficient (i.e. positive or negative)

© 2026 Cook A et al. *JAMA Network Open*.

eReferences.

1. Folgori L, Tersigni C, Hsia Y, Kortsalioudaki C, Heath P, Sharland M, et al. The relationship between Gram-negative colonization and bloodstream infections in neonates: a systematic review and meta-analysis. Clinical Microbiology and Infection. 2018;24(3):251-7.
2. Williams P, Putri ND, Harrison J, Dickson B, Lestari D, Ramsay J, et al. Delineating the risk factors, sources of acquisition and clinical outcomes in neonates colonised with multidrug-resistant bacteria: The NeoCol study. 2026.
3. Kagia N, Kosgei P, Ooko M, Wafula L, Mturi N, Anampiu K, et al. Carriage and Acquisition of Extended-spectrum beta-Lactamase-producing Enterobacterales Among Neonates Admitted to Hospital in Kilifi, Kenya. Clin Infect Dis. 2019;69(5):751-9.
4. Beaumont AL, de Lauzanne A, Criscuolo A, Fabre L, Rabenandrasana MAN, Randriamanga NF, et al. Contribution of maternal gut carriage to neonatal acquisition of extended-spectrum beta-lactamase-producing Enterobacterales in Madagascar and Cambodia. Nat Commun. 2025;16(1):10399.
5. Rettedal S, Löhr IH, Bernhoff E, Natås OB, Sundsfjord A, Øymar K. Extended-spectrum β-lactamase-producing Enterobacteriaceae among pregnant women in Norway: prevalence and maternal–neonatal transmission. Journal of Perinatology. 2015;35(11):907-12.
6. Philipp Foessleitner, Julia Gasser, Herbert Kiss, Albert Flunt, Elisabeth Presterl, Ljubomir Petricevic, et al. Vaginal colonization of extended-spectrum beta-lactamase-producing bacteria during pregnancy: an observational cohort study. European Journal of Obstetrics & Gynecology and Reproductive Biology. 2020;246:86-9.
7. Minotti C, Jost K, Aghlmandi S, Schlaeppi C, Sieswerda E, Van Werkhoven CH, et al. All-cause mortality and infection-related outcomes of hospital-initiated kangaroo care versus conventional neonatal care for low-birthweight infants: a systematic review and meta-analysis. The Lancet Child & Adolescent Health. 2025;9(7):470-83.

© 2026 Cook A et al. *JAMA Network Open*.
