# Supplementary material for: Neonatal Unit Colonization Pressure and Acquisition of Extended-Spectrum β-Lactamase Gut Colonization
Source: JAMA Netw Open. 2026 Sep 15;9(9):e2632644. doi: 10.1001/jamanetworkopen.2026.32644 (PMC13579436; doi:10.1001/jamanetworkopen.2026.32644)
Supplement: Supplementary file 2 [file jamanetwopen-e2632644-s002.pdf]

# Data Sharing Statement

Cook. The Association Between Neonatal Unit Colonization Pressure and Acquisition of Extended-Spectrum β-Lactamase Gut Colonization. *JAMA Netw Open*. Published September 11, 2026. doi:10.1001/jamanetworkopen.2026.32644

**Data**
**Data available:** No

**Additional Information**
**Explanation for why data not available:** Data can be made available for specific research questions with approval from the study Sponsor.
